# Role of β-hydroxybutyrate, its polymer poly-β-hydroxybutyrate and inorganic polyphosphate in mammalian health and disease

**DOI:** 10.3389/fphys.2014.00260

**Published:** 2014-07-17

**Authors:** Elena N. Dedkova, Lothar A. Blatter

**Affiliations:** Department of Molecular Biophysics and Physiology, Rush University Medical CenterChicago, IL, USA

**Keywords:** inorganic polyphosphate, β-hydroxybutyrate, poly-β-hydroxybutyrate, cardiovascular disease, heart failure, mitochondrial permeability transition pore

## Abstract

We provide a comprehensive review of the role of β-hydroxybutyrate (β-OHB), its linear polymer poly-β-hydroxybutyrate (PHB), and inorganic polyphosphate (polyP) in mammalian health and disease. β-OHB is a metabolic intermediate that constitutes 70% of ketone bodies produced during ketosis. Although ketosis has been generally considered as an unfavorable pathological state (e.g., diabetic ketoacidosis in type-1 diabetes mellitus), it has been suggested that induction of mild hyperketonemia may have certain therapeutic benefits. β-OHB is synthesized in the liver from acetyl-CoA by β-OHB dehydrogenase and can be used as alternative energy source. Elevated levels of PHB are associated with pathological states. In humans, short-chain, complexed PHB (cPHB) is found in a wide variety of tissues and in atherosclerotic plaques. Plasma cPHB concentrations correlate strongly with atherogenic lipid profiles, and PHB tissue levels are elevated in type-1 diabetic animals. However, little is known about mechanisms of PHB action especially in the heart. In contrast to β-OHB, PHB is a water-insoluble, amphiphilic polymer that has high intrinsic viscosity and salt-solvating properties. cPHB can form non-specific ion channels in planar lipid bilayers and liposomes. PHB can form complexes with polyP and Ca^2+^ which increases membrane permeability. The biological roles played by polyP, a ubiquitous phosphate polymer with ATP-like bonds, have been most extensively studied in prokaryotes, however polyP has recently been linked to a variety of functions in mammalian cells, including blood coagulation, regulation of enzyme activity in cancer cells, cell proliferation, apoptosis and mitochondrial ion transport and energy metabolism. Recent evidence suggests that polyP is a potent activator of the mitochondrial permeability transition pore in cardiomyocytes and may represent a hitherto unrecognized key structural and functional component of the mitochondrial membrane system.

## What is β-hydroxybutyrate?

**Figure 1:**
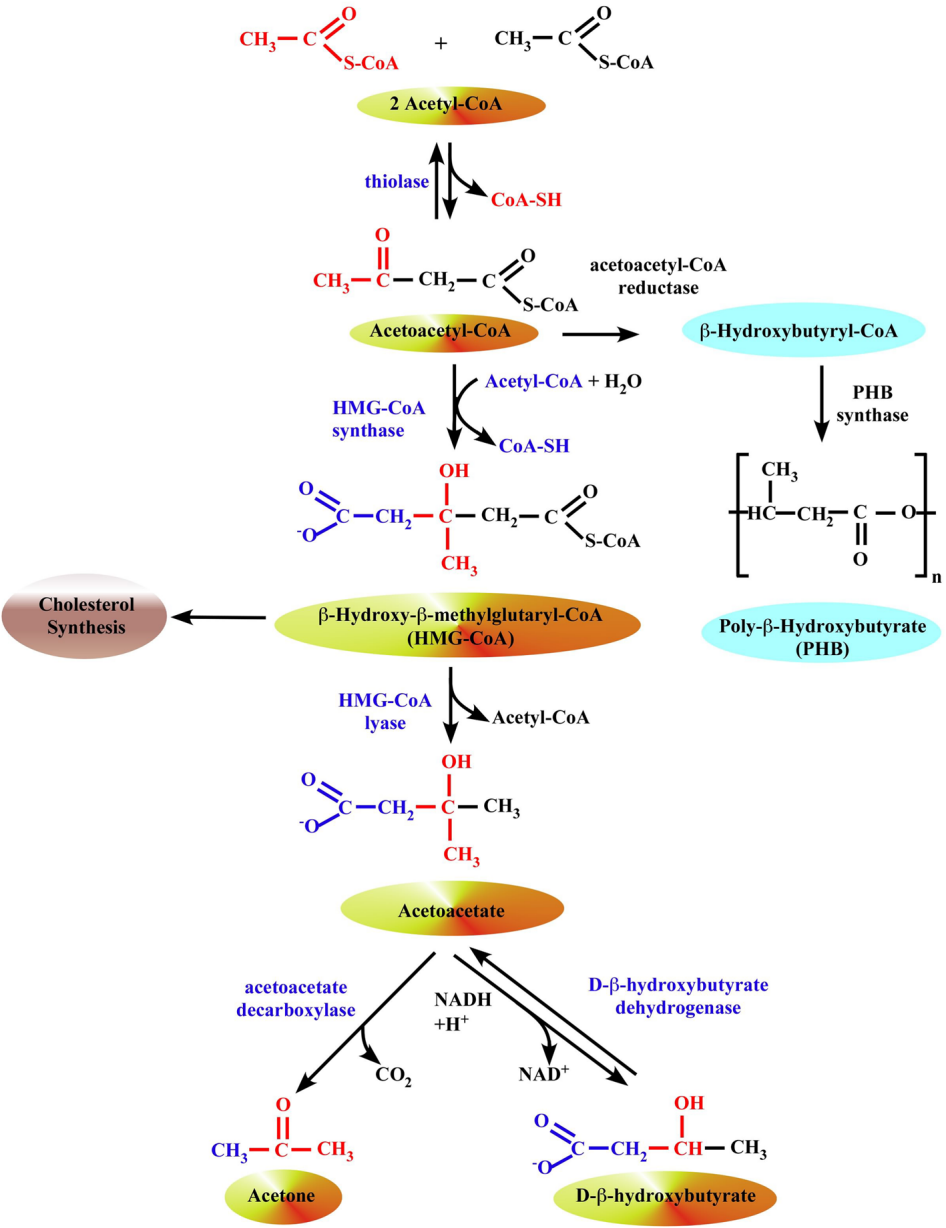
**Formation of ketone bodies in liver mitochondria**. The synthesis of β-OHB begins with the condensation of two molecules of acetyl-CoA to form acetoacetyl-CoA, the parent of the three ketone bodies, by a ketothiolase enzyme. In prokaryotes, this intermediate is subsequently reduced with NADPH to hydroxybutyryl-CoA by acetoacetyl-CoA reductase, and hydroxybutyryl-CoA may then be polymerized to form PHB by the enzyme PHB synthase. In eukaryotes, 3-hydroxy-3-methylglutaryl-CoA synthase (HMG synthase) catalyzes the condensation of acetoacetyl-CoA with a third acetyl-CoA to form β-hydroxy-β-methylglutaryl-CoA (HMG-CoA). The enzyme HMG-CoA lyase then catalyzes the decomposition of HMG-CoA to form acetoacetate and acetyl-CoA, and acetoacetate is further reduced with NADH by phosphatidylcholine-dependent mitochondrial D-β-hydroxybutyrate dehydrogenase to form β-OHB (Lehninger et al., 1960; Marks et al., 1992). Acetoacetate is also non-enzymatically decarboxylated to acetone.

**Figure 2:**
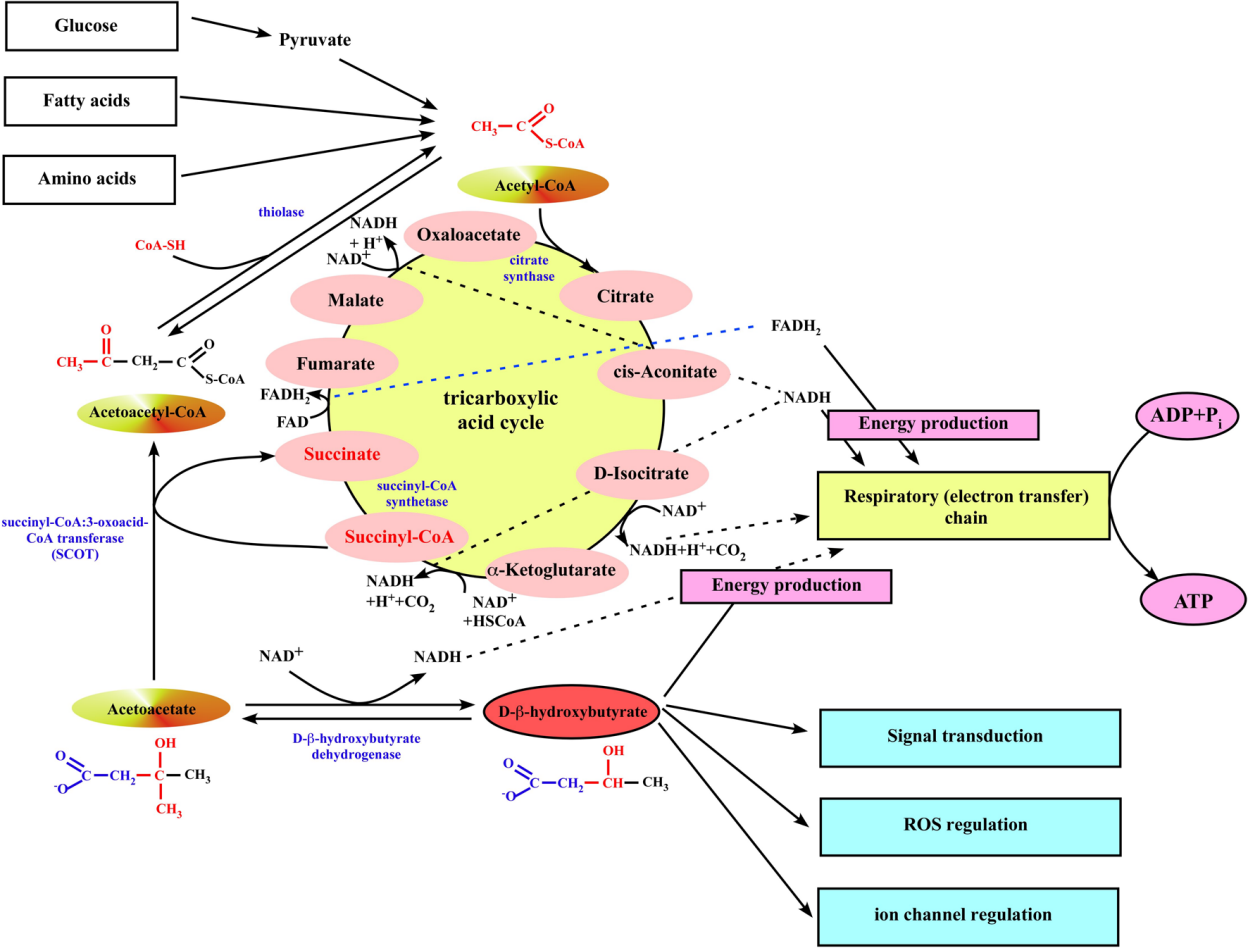
**Ketone body utilization in mitochondria of extrahepatic organs**. When ketone bodies are delivered to the peripheral organs, β-OHB is oxidized back to acetoacetate by the mitochondrial BDH1. Then, acetoacetate gets converted to acetoacetyl-CoA by the mitochondrial enzyme succinyl-CoA:3-oxoacid-CoA transferase (SCOT). The succinyl-CoA:3-oxoacid-CoA transferase uses succinyl-CoA as the CoA donor, forming succinate and acetoacetyl-CoA. This reaction bypasses the succinyl-CoA synthetase step of the TCA cycle, although it does not alter the amount of carbon in the cycle. Next, mitochondrial thiolase cleaves the acetoacetyl-CoA into two molecules of acetyl-CoA, which can generate energy by entering the TCA cycle pathway.

β-Hydroxybutyrate (β-OHB; also known as 3-hydroxybutyric acid) is a metabolic intermediate that constitutes ~70% of ketone bodies produced in liver mitochondria mainly from the oxidation of fatty acids released from adipose tissue (Persson, 1970). The term “ketone bodies” refers to three molecules: (1) β-OHB, (2) its dehydrogenated counterpart acetoacetate (AcAc), and (3) the decarboxylated AcAc, acetone (Figure 1). Acetone, produced in smaller quantities than the other ketone bodies, is exhaled and essentially is unmeasurable in healthy individual (Laffel, 1999; Cahill and Veech, 2003). AcAc and β-OHB are transported by blood to the extrahepatic tissues, where they are oxidized via the tricarboxylic acid (TCA) cycle to provide the energy required by tissues such as skeletal and heart muscle and the renal cortex (Figure 2). In the normal adult heart, mitochondrial oxidative phosphorylation provides more than 95% of the ATP generated for its mechanical, electrical and homeostatic needs. Fatty acid oxidation accounts for up to 70% of the ATP produced by the heart, with metabolism of glucose, lactate, amino acids and ketone bodies supplying the rest (reviewed in Lopaschuk et al., 2010; Cotter et al., 2013). However, ketone body contribution to the overall energy metabolism in the heart and other extrahepatic tissues increases significantly after prolonged exercise, during fasting, adherence to low carbohydrate ketogenic diet (LCKD) and in the neonatal period (Veech et al., 2001; Veech, 2004). In these physiological situations, the concentration of circulating blood β-OHB rises from ~0.1 mM observed in normal fed state to ~1 mM after few hours of fasting, and up to 5–7 mM after prolonged starvation (Cahill, 1970; Robinson and Williamson, 1980; Laffel, 1999; Cahill and Veech, 2003). If the release of free fatty acids from adipose tissue exceeds the capacity of tissue to metabolize them, as occurs during insulin deficiency of type I diabetes or less commonly in the insulin-resistant of type II diabetes, severe and potentially fatal diabetic ketoacidosis can occur where blood β-OHB levels can reach up to 25 mM (Lebovitz, 1995; Cahill and Veech, 2003). Mild elevation of blood ketone bodies also occurs during the process of normal aging (Sengupta et al., 2010) and during congestive heart failure (HF) (Kupari et al., 1995; Lommi et al., 1996, 1997) however it remains unclear whether this elevation represents an adaptive mechanism required to maintain cell metabolism or actually contributes to the progression of disease. Induction of mild states of hyperketonemia may have certain therapeutic benefits (Veech et al., 2001; Veech, 2004; Clarke et al., 2012b). Ketone body oxidation is especially critical in the brain which cannot utilize fatty acids for energy (free fatty acids do not cross the blood-brain barrier) when blood glucose levels become compromised. In this case, ketone bodies provide the brain with an alternative source of energy, amounting to nearly 75% of the brain's energy needs during periods of prolonged fasting and starvation (Owen et al., 1967; Cahill, 2006). It has been known for many years that children with multidrug-resistant refractory epilepsy improve dramatically on a strict LCKD (Veech, 2004), and recent data indicate that β-OHB supplementation protects neurons in models of Alzheimer and Parkinson's disease (Kashiwaya et al., 2000; Tieu et al., 2003; Reger et al., 2004). The laboratory of Veech (Kashiwaya et al., 1994; Sato et al., 1995) was first to report that addition of 4 mM β-OHB to the working perfused rat heart induced an increase in work output but a significant decrease in oxygen consumption. The data demonstrated that the increased efficiency was the result of the widening mitochondrial substrate ratio of NADH and NAD^+^ between complex 1 and complex 2 of the mitochondrial respiratory chain. The net effect is a greater potential for ATP production making β-OHB the most efficient fuel in the heart.

Evolutionary, metabolism of ketone bodies is conserved among eukarya, bacteria, and archaea (Reusch, 1992; Aneja et al., 2002; Cahill, 2006). Most bacteria use poly-β-hydroxybutyrate (PHB, Figure 1), a polymerized form of β-OHB, as an energy store (Anderson and Dawes, 1990; Reusch, 1992, 2012). PHB consists up to 90% of dry weight in some protozoans. Even archaea use it for energy storage, which suggests it has been around for well over 2–3 billion years. It is possible that its selection was aided by the periods of low environmental oxygen that occurred during the Archaean, Proterozoic, and Palaeozoic eras (Cahill, 2006). PHB is stored as several large granules in the cytoplasm (Anderson and Dawes, 1990; Reusch, 2012), therefore having very little osmotic effect which is in contrast to two other fundamental archaeal energy stores, inorganic polyphosphate (polyP) (Kornberg, 1995; Kornberg et al., 1999) and various polysaccharides (Dawes and Senior, 1973). It has been believed that both polyP and PHB remained only in the prokaryotes, however the raising number of studies demonstrated the presence of both polyP and PHB in mammalian cells, and their important physiological roles. Since only small amounts of PHB and polyP are detected in mammalian cells, it is thought that they do not serve as an energy store (Kulaev et al., 2004) but rather participate in cell signaling and proliferation, regulation of mitochondrial function, channel activity, blood coagulation and inflammation, and bone tissue development. This review discusses the roles of β-OHB, PHB, and inorganic polyP with specific focus on cardiovascular health and disease.

## What is poly-β-hydroxybutyrate (PHB)?

PHB (also known as Poly-(*R*)-3-hydroxybutyrate) is a biopolymer consisting of linear chains of β-OHB (Figure 1). Three types of PHB with different numbers of β-OHB units and with different functions have been discovered: (i) high molecular weight storage PHB consists of 10,000 to >1,000,000 β-OHB residues (storage PHB), (ii) low molecular weight PHB with medium–chain length consisting of 100–300 residues (oligo-PHB), and (iii) short-chain conjugated PHB (cPHB) in which low numbers of β-OHB residues (≤30) are covalently linked to proteins (see Reusch, 1992, 2012, 2013 for comprehensive reviews). Storage PHB was first discovered in granular inclusion bodies (termed carbonosomes) within the cytoplasm of *Bacillus megaterium* in 1925, and later in a wide variety of archaea and eubacteria, principally those that inhabit soil and water ecosystems (Nuti et al., 1972; Anderson and Dawes, 1990; Poli et al., 2011). PHB is produced by these prokaryotes when carbon sources are freely available but other nutrients are limited, thus PHB is considered to serve as a carbon and energy store in these organisms. PHB located within carbonosomes are covered by a layer of lipids and proteins, which include enzymes involved in PHB synthesis and degradation (Potter and Steinbuchel, 2005; Rehm, 2006; Jendrossek, 2009), have attained considerable commercial importance as ingredients of biodegradable plastics and high-technology materials in the medical field (Wu et al., 2009). PHB of medium-chain length was first discovered in the cytoplasmic membranes of genetically-competent bacteria *Azotobacter vinelandii, Bacillus subtilis* and *Haemophilus influenzae* by Reusch and Sadoff (1983) and later in *Escherichia coli (E. Coli)* (Reusch and Sadoff, 1983; Reusch et al., 1986). Interestingly, that *H. influenzae* and *E. coli* do not accumulate PHB granules. Medium-chain PHB, like storage PHB, is insoluble in water and soluble in chloroform, and non-covalently associated with other molecules. In 1989, medium-chain length PHB was recovered from membranes, mitochondria and microsomes of eukaryotes (Reusch, 1989), and in 1992 from very low density and low density lipoproteins (VLDL and LDL) of human plasma (Reusch et al., 1992). The identity of medium-chain length PHB in representative prokaryotic and eukaryotic organisms was revealed by ^1^H-NMR spectroscopy by Reusch (1992) and confirmed by Seebach et al. (1994). Medium-chain length PHB has been found associated with inorganic polyP in non-covalent complexes that are postulated to play a role in transbilayer transport of cations (Reusch and Sadoff, 1988; Reusch et al., 1995; Das et al., 1997) and deoxyribonucleic acids (Castuma et al., 1995; Huang and Reusch, 1995; Reusch, 2000). Huang and Reusch (1996) discovered short-chain PHB (≤10 residues) covalently bound to specific proteins in the membranes and the cytoplasm of *E. coli*. It is generally thought to consist of ≤30 residues but this remains unclear due to the lability of the ester bond and the paucity of samples examined to date. Unlike storage PHB that is segregated within cytoplasmic granules and medium-chain PHB that is “dissolved” in lipid environments, cPHB has been found in all cell compartments of prokaryotes and eukaryotes and in intracellular fluids (Reusch et al., 1997, 2002, 2003; Reusch, 1999; Norris et al., 2009). Indeed, the majority of PHB in cells that do not accumulate long-chain storage PHB is cPHB. It has been postulated that cPHB forms supra-molecular complexes with proteins via covalent bonds and multiple hydrophobic interaction sites (Reusch, 1989, 1999; Seebach et al., 1994). The physical properties of cPHB and large size of the polyester may have substantial structural and functional impacts on the protein. The high-energy C-terminal CoA-ester group, derived from PHB metabolic precursors, presumably acts as a cofactor for the enzymatic reaction in which a covalent bond to the protein is formed (Zhang et al., 2011). This covalent attachment of cPHB is known as PHBylation (Cao et al., 2013) and plays roles in protein folding, protein sorting, or retention of inorganic polyP (Xian et al., 2007; Negoda et al., 2010; Cao et al., 2013). In eukaryotes, cPHB was found to be bound to the Ca^2+^-ATPase pump of human erythrocyte membranes (Reusch et al., 1997), which is the sole transporter of Ca^2+^ in red blood cells. Interestingly, inorganic polyP was also present in this complex, and it was suggested that Ca^2+^-ATPase is a supra-molecular structure consisting of protein, cPHB, and polyP. Solvation of polyP by PHB could allow this polyanion to penetrate into the bilayer portion of Ca^2+^-ATPase, and possibly mediate Ca^2+^ transfer (Reusch et al., 1997). Recently, it has been found that a member of the transient receptor potential channel family of the melastatin subgroup, TRPM8, which is a major sensor for cold temperatures in the peripheral nervous system, is significantly modified by both cPHB and polyP (Zakharian et al., 2009, 2010; Cao et al., 2013). Moreover, it has been shown that cPHB/polyP complexes isolated from rat liver mitochondria can form voltage-dependent ion channels with multiple conductance states when incorporated in planar lipid bilayers (Pavlov et al., 2005b). The characteristics of this channel activity closely resembled the behavior of the mitochondrial permeability transition pore (mPTP) channel observed in patch-clamp experiments in native mitoplasts (see below for more information). The ubiquitous occurrence of the medium-chain and short-chain polyester (Reusch and Sadoff, 1988; Reusch, 1989, 1992; Reusch et al., 1992, 2003; Seebach et al., 1994; Norris et al., 2009; Zakharian et al., 2009, 2010; Elustondo et al., 2012), suggests that PHB, like polyisoprenoids, polypeptides, polysaccharides, and polynucleotides, is a fundamental constituent of biological cells.

## Mechanisms of β-OHB/PHB production and utilization

Ketone body metabolism includes both ketogenesis and ketolysis (see McGarry and Foster, 1980; Robinson and Williamson, 1980; Cotter et al., 2013 for comprehensive reviews). Ketogenesis is the process by which fatty acids are transformed into AcAc and β-OHB (Figures 1, 2). This process takes place in the mitochondria of liver cells and can occur in response to unavailability of blood glucose (Robinson and Williamson, 1980). The production of ketone bodies is then initiated to make available energy primarily from fatty acids. Fatty acids are enzymatically broken down in β-oxidation to form acetyl-CoA. Under normal conditions, acetyl-CoA is further oxidized and its energy transferred as electrons to NADH and FADH_2_ in the TCA cycle, and further to ATP in the mitochondrial respiratory chain (Figure 2). However, if the amounts of acetyl-CoA generated in fatty acid β-oxidation challenge the processing capacity of the TCA cycle or if activity in the TCA cycle is low due to low amounts of intermediates such as oxaloacetate, acetyl-CoA is then used instead in biosynthesis of ketone bodies via acetoacyl-CoA and β-hydroxy-β-methylglutaryl-CoA (HMG-CoA). Deaminated amino acids that are ketogenic, such as leucine, also feed the TCA cycle, forming AcAc and acetyl-CoA, and thereby generate up to 4% of circulating ketones (Merritt et al., 2011). Glucose metabolism accounts for ~1% of circulating ketones in states of low-carbohydrate intake because pyruvate predominantly enters the hepatic TCA cycle via carboxylation to oxaloacetate or malate rather than decarboxylation (to acetyl-CoA) (Magnusson et al., 1991; Merritt et al., 2011; Jeoung et al., 2012).

The synthesis of β-OHB begins with (i) the condensation of two molecules of acetyl-CoA to form acetoacetyl-CoA by a ketothiolase enzyme; this is simply the reversal of the last step of β–oxidation (Figure 1). In prokaryotes, this intermediate is subsequently reduced with NADPH to hydroxybutyryl-CoA by acetoacetyl-CoA reductase, and hydroxybutyryl-CoA may then be polymerized to form PHB by the enzyme PHB synthase (Anderson and Dawes, 1990; Poli et al., 2011; Reusch, 2013). (ii) In eukaryotes, 3-hydroxy-3-methylglutaryl-CoA synthase 2 (HMG synthase 2) catalyzes the condensation of acetoacetyl-CoA with a third acetyl-CoA to form HMG-CoA (Bahnson, 2004). HMG synthase 2 is exclusively present in liver mitochondria. HMG synthase 1 is located in cytosol and associated with cholesterol biosynthesis. (iii) The enzyme HMG-CoA lyase then catalyzes the decomposition of HMG-CoA to form AcAc and acetyl-CoA, and AcAc is further reduced with NADH by phosphatidylcholine-dependent mitochondrial D-β-hydroxybutyrate dehydrogenase to form β-OHB (Lehninger et al., 1960; Marks et al., 1992). AcAc is also non-enzymatically decarboxylated to acetone. The ratio of β-OHB to AcAc depends on the NADH/NAD^+^ ratio inside mitochondria; if NADH concentration is high, the liver releases a higher proportion of β-OHB. Enzymes that polymerize β-OHB or its CoA ester have not yet been identified in eukaryotes (Reusch, 2013). Ketone bodies are released by the liver via solute carrier 16A (SLC16A) family members 1, 6, and 7 and circulate to extrahepatic tissues where they primarily undergo terminal oxidation (Halestrap, 2012; Halestrap and Wilson, 2012; Hugo et al., 2012).

When ketone bodies are delivered to the peripheral organs, β-OHB is oxidized back to AcAc by the mitochondrial D-β-hydroxybutyrate dehydrogenase (Figure 2). The utilization of ketone bodies requires an enzyme not present in the ketone body biosynthetic pathway, succinyl-CoA:3-oxoacid-CoA transferase (also known as SCOT), which converts AcAc to acetoacetyl-CoA. This enzyme is not present in liver, and therefore the liver lacks the ability to utilize ketones. The SCOT uses succinyl-CoA in the TCA cycle as the CoA donor, forming succinate and acetoacetyl-CoA. This reaction bypasses the succinyl-CoA synthetase step of the TCA cycle, although it does not alter the amount of carbon in the cycle. Next, mitochondrial thiolase cleaves the acetoacetyl-CoA into two molecules of acetyl-CoA, which can generate energy by entering the TCA cycle pathway (Figure 2). This also implies that TCA cycle must be running to allow ketone body utilization.

## Feedback regulation of β-OHB synthesis via β-OHB receptors

β-OHB is a ligand for at least two G-protein-coupled receptors (GPCRs) that bind short-chain fatty acids. HCAR2 (hydroxycarboxylic acid receptor 2; also known as PUMA-G or Gpr109), a G_i/o_-coupled GPCR, first identified as a nicotinic acid receptor (Tunaru et al., 2003), was recently shown to bind and be activated by β-OHB (Taggart et al., 2005). It has been demonstrated that fatty acid derived β-OHB specifically activates HCAR2 receptors within physiologically relevant β-OHB concentrations (K_i_ = 0.7 mM) (Taggart et al., 2005), typically observed in serum during short-term fasting. Like nicotinic acid, β-OHB reduces lipolysis in mouse adipocytes (with EC_50_ ~2 mM) possibly creating a negative feedback mechanism to regulate availability of the fatty acid precursors of ketone body metabolism (Taggart et al., 2005; Offermanns et al., 2011). Indeed, in a study of the serum free fatty acids-lowering effect of β-OHB infused in humans, Senior and Loridan (1968) proposed that during starvation ketone bodies exert “a fine regulatory adjustment” of their own synthesis by inhibiting adipocyte lipolysis. β-OHB also binds to and antagonizes the free fatty acid receptor 3 (FFAR3, also known as GPR41), another G_i/o_ protein-coupled receptor that is present in sympathetic ganglions, thereby suppressing sympathetic activity and, in turn, overall metabolic rate in mice (Kimura et al., 2011; Won et al., 2013). Thus, through its actions on HCAR2 and FFAR3, β-OHB may reduce lipolysis, reduce sympathetic tone, and lower metabolic rate.

## Role of β-OHB in cardiovascular health and disease

The rate of fatty acid oxidation in the normal healthy heart is a function of the arterial free fatty acid concentration and the activities of the enzymes involved in fatty acid transport and oxidation in the mitochondria, specifically carnitine-palmitoyl transferase I (CPT-I) and the enzymes of the β-oxidation pathway (Lopaschuk et al., 1994, 2010; Kunau et al., 1995). The heart readily oxidizes ketone bodies (β-OHB and AcAc) in a concentration-dependent manner at the expense of fatty acid oxidation (Lammerant et al., 1985; Forsey et al., 1987; Stanley et al., 2003). β-OHB has the ability to inhibit lipolysis (Hron et al., 1978), thereby inhibiting the production of the free fatty acid that is implicated in extending myocardial injury by elevating the expression of cardiac mitochondrial uncoupling proteins and decreasing the expression of glucose transporter 4 (Murray et al., 2004; Opie, 2004). In fact, the reduction of concentrations of free fatty acid concentration and the use of alternative substrates was proposed for treatment of patients with HF (Murray et al., 2004). This treatment would reduce mitochondrial uncoupling and restore glucose uptake, thereby improving cardiac efficiency without a fall in cardiac work. In addition, inhibition of fatty acid utilization reduces oxygen demand of adjacent normal myocardial tissue (Lammerant et al., 1985), preventing the extent of cellular damage (Liedtke et al., 1982). Thus, elevated concentrations of β-OHB may prevent myocardial damage by preventing the formation of damaging intermediates as well as by serving as an alternate energy source (Figure 2). Indeed, cardioprotective effects have been observed using *in vivo* ischemia/reperfusion approaches in rats subjected to starvation-induced ketosis, initiated through prolonged fasting, and also via intravenous injection of β-OHB immediately prior to ischemic injury, which conferred a significant decrease in both infarct size and myocardial cell death (Zou et al., 2002; Snorek et al., 2012). Moreover, it has been demonstrated that LCKD enhances cardiac tolerance to global ischemia (Al-Zaid et al., 2007). This study revealed a significant decrease in the number of mitochondria in rats fed a high carbohydrate diet and an increase in the number of mitochondria in those fed a LCKD compared to normal diet group. Rats on LCKD had a remarkable tolerance to ischemia and a faster recovery of cardiac function following reperfusion (Al-Zaid et al., 2007). Transcriptional upregulation of key mediators of mitochondrial oxidative phosphorylation by LCKD significantly extended the lifespan of mice with *Med30^zg^* mutation (Krebs et al., 2011). Typically, the *Med30^zg^* mutation causes a progressive and selective decline in the transcription of genes necessary for oxidative phosphorylation and mitochondrial integrity, eventually leading to cardiac failure. A ketogenic diet favorably affected serum biomarkers for cardiovascular disease in normal-weight men (Al-Zaid et al., 2007) and in obese diabetic subjects (Dashti et al., 2007). Cardioprotective effects of β-OHB could be also related to their ability to suppress oxidative stress via transcriptional (Krebs et al., 2011; Shimazu et al., 2013) regulation of key mediators of oxidative stress. The recent study from Eric Verdin's group (Shimazu et al., 2013) demonstrated that β-OHB is an endogenous and specific inhibitor of class I histone deacetylases (HDACs). Inhibition of HDAC by β-OHB was correlated with global changes in transcription, including that of the genes encoding oxidative stress resistance factors Forkhead box O3a (FOXO3a) and MT2. Consistent with increased FOXO3a and MT2 activity, treatment of mice with β-OHB led to substantial protection against oxidative stress. The anti-oxidant effect of β-OHB could be also related to the ability of ketone bodies to oxidize co-enzyme Q (Sato et al., 1995; Veech et al., 2001; Veech, 2004). The major source of mitochondrial free radicals is the half-reduced semiquinone of co-enzyme Q (Chance et al., 1979). Q semiquinine reacts directly with O_2_ to form the superoxide radical O^2−^. By decreasing the reduced form of co-enzyme Q, the mitochondrial production of free radical can be decreased. In a second action of ketone body metabolism, in addition to reducing the mitochondrial NAD^+^/NADH redox couple, there is also a reduction of the cytoplasmic free NADP^+^/NADPH couple. This favors the reduction of glutathione, which is near equilibrium through the action of glutathione reductase (Krebs and Veech, 1969). This is turn would favor the destruction of H_2_O_2_ by the glutathione peroxidase reaction (Veech et al., 2001; Squires et al., 2003). However, glutathione levels were unchanged during glutamate-induced ROS generation in neurons while ketones inhibited ROS generation by increasing NADH oxidation (Haynes et al., 2003).

While there are many studies which demonstrate that a ketogenic diet results in the improvement of cardiovascular health and significant weight loss, completely opposite results were also reported. For example in an animal study by Wang et al. (2008), rats were fed with LCKD or control diet for 2 weeks and isolated hearts were subjected to normal perfusion in Langendorff mode, with 30 min global low flow ischemia (LFI) followed by 60 min reperfusion, or 60 min LFI followed by 120 min reperfusion. They found that LCKD diet resulted in impaired left ventricular performance during global LFI, reduced recovery of function following LFI and reperfusion, and 10- to 20-fold increased injury as measured by lactate dehydrogenase release and histologic infarct area. Serum FFA, glucose and lactate levels were not different between diet groups, but LCKD did lead to a 2-fold increase in β-OHB (from 0.3 to 0.6 mM) and a 50% decrease in the fed-state insulin level (from 34 to 15 μU/ml) compared to control.

In addition, ketogenic diets can cause biochemical disturbances and cardiac dysfunction in certain vulnerable patients, possibly due to latent defects in ketone body metabolism (Best et al., 2000). In this case study, 20 patients on a ketogenic diet as a treatment for partial seizures and refractory childhood epilepsy were investigated. Prolonged QT interval [with corrected QT (QTc) longer than 450 ms] and cardiac chamber enlargement were found in three pediatric patients (15%). There was a significant correlation between prolonged QTc and both low serum bicarbonate and high β-OHB levels suggesting that the levels of acidosis or ketosis may be important factors in these cardiovascular complications. However, it is important to emphasize that in 17 patients who actually benefited from ketogenic diet (these patients were weaned off anticonvulsants), the serum levels of β-OHB ranged between 4.4 and 6.9 mmol/L (Best et al., 2000). In one patient presented with cardiovascular complications, the serum level of β-OHB of 11.7 mmol/L was detected on admission to the hospital. This excessive elevation of β-OHB definitely can play a role in the development of cardiovascular complications. Cardiovascular disease is a well-known complication of diabetes where levels of β-OHB could reach 25 mM or more in poorly controlled type 1 diabetes (Cahill and Veech, 2003). Age may also play a role, as younger children can have higher β-OHB levels. Some epilepsy patients may have abnormal transcellular membrane ion channels resulting in a predisposition to develop conduction abnormalities as seen in those who develop long QT on cisapride, tricyclics, or class I and III antiarrhythmics (Best et al., 2000). One study revealed that β-OHB at high concentrations (10 mM) reduced L-type Ca^2+^ current in guinea pig ventricular myocytes but only in the presence of the β–adrenergic agonist isoproterenol, however it had no effect on L-type Ca^2+^ current under basal conditions (Kurihara et al., 2012). Another study suggested that the L-stereoisomer of β-OHB could block the transient outward K^+^ current (*I_to_*) in murine ventricular myocytes causing action potential prolongation (Doepner et al., 1997) while D-β-OHB had no effect. However, this study was later retracted and no other studies are currently available demonstrating possible effects of β-OHB on channel activity in cardiomyocytes. Moreover, hepatic ketogenesis only produces D-β-OHB, which is the only form that is a D-β-hydroxybutyrate dehydrogenase substrate, and thus, a substrate for oxidation (Scofield et al., 1982). L-β-OHB is possibly formed during hydrolysis of the β-oxidation intermediate L-β-OHB-CoA in cardiac mitochondria and does not circulate in the blood (Scofield et al., 1982; Tsai et al., 2006). Interestingly, only the heart contains significant amounts of L-stereoisomer of β-OHB (Tsai et al., 2006) but its physiological role is unclear. It has been shown that only D-β-OHB was able to inhibit glucose utilization in cardiomyocytes while L-stereoisomer had no effect (Tsai et al., 2006). In a concentration of 5 mM of D-β-OHB, a maximum inhibition effect of 61% of control was found, but L-β-OHB did not interfere with glucose utilization in concentrations from 0.5 to 5 mM. Additionally, when cells were in medium containing L-β-OHB and 5 mM of D-β-OHB, the reduced glucose utilization caused by D-β-OHB gradually recovered with increasing concentrations of L-β-OHB (Tsai et al., 2006).

Currently, very limited data is available on the role of ketone body metabolism in HF. While a mild elevation of blood ketone bodies occurs during congestive HF (Kupari et al., 1995; Lommi et al., 1996, 1997), no changes in cardiac ketone body utilization were detected in humans with advanced HF (Janardhan et al., 2011). It remains unclear whether this elevation represents an adaptive mechanism required to maintain cell metabolism in conditions of HF or actually contributes to the progression of disease. It has been suggested that ketone bodies might be an important fuel during persistent human atrial fibrillation (AF) (Mayr et al., 2008). Proteomic and metabolic analysis of atrial tissue harvested during cardiac surgeries from patients with AF revealed a 2-fold increase in SCOT expression, a mitochondrial matrix enzyme required to activate ketones by transferring CoA from succinyl-CoA to AcAc, the key reaction in ketolytic energy production (see Figure 2). Consistent with the proteomic findings, metabolomic analysis revealed a rise in the levels of β-OHB and ketogenic amino acids, notably tyrosine, which forms AcAc and fumarate during catabolism. Fumarate levels were markedly elevated in persistent AF, and consequently the fumarate/succinate ratio increased. The latter is often used as an indicator for the redox state of the coenzyme Q couple (Sato et al., 1995), which is the cofactor for the succinate dehydrogenase reaction and links complex I and complex III of the respiratory chain via its redox span. Both NADH dehydrogenase (complex I) and ubiquinol cytochrome C reductase (complex III) were among the differentially expressed proteins in the proteomic screen. Moreover, animal studies have shown that administration of glucose plus ketone bodies resulted in an increase in the fumarate/succinate ratio similar to the observation in humans with AF (Sato et al., 1995). Interestingly, that elevated myocardial energy expenditure in patients with HF was associated with significant changes in serum metabolomics profiles, especially the concentration of β-OHB, acetone and succinate (Du et al., 2014). Elevated myocardial energy expenditure correlates with reduced left ventricular ejection fraction in HF, and has also been documented as an independent predictor of cardiovascular mortality (Palmieri et al., 2008). Again, it was not investigated whether this elevation in ketone bodies and succinate was a result of adaptation during cardiac remodeling in conditions of HF or represents a maladaptive mechanism contributing to the development of the disease. Definitely, more studies are required to shed light on the role of ketone bodies in cardiovascular diseases. Moreover, to avoid the negative side effects of the ketogenic diets, absorbable ketone body esters comprised of the mono ester of D-β-OHB and R-1,3-butanediol were developed by Veech and colleagues (Clarke et al., 2012a,b). It has been demonstrated that feeding ketone body esters to rats lowers blood glucose and insulin, demonstrating that ketosis actually increases insulin sensitivity (Veech, 2013, 2014). It was suggested that the negative effects of LCKD were associated not with ketone bodies themselves but rather with enhanced release of free fatty acids from the adipose tissue and the correspondent increase in peroxisome proliferators-activated receptor (PPAR) transcription factors with many undesirable effects like development of the atherosclerosis as one example (Kersten et al., 2000).

## Roles of cPHB in health and disease

Elevated levels of plasma β-OHB observed in HF and diabetes could potentially lead to increased accumulation of cPHB. The pioneering work of Dr. Rosetta Reusch from Michigan State University demonstrated that cPHB could bind to a wide range of proteins modifying the function of voltage-gated ion channels and calcium ATPase pumps (see above and Reusch, 1989, 1992). Dr. Reusch has further demonstrated the importance of cPHB to medicine by showing that cPHB is present in a wide variety of human tissues and also in atherosclerotic plaques (Reusch, 1989, 1992; Reusch et al., 1992; Seebach et al., 1994). However, during digestion processes cPHB may become detached from proteins, and its physical properties—water-insoluble, high intrinsic viscosity, ability to make bilayers non-selectively permeant to ions, sticky (forms multiple non-covalent bonds)—suggest that it may be a factor in the development of some human and animal diseases (Reusch, 1992). Reusch et al. (2003) compared cPHB levels in plasma and tissues of streptozotocin diabetic rats with those in healthy Sprague–Dawley rats. They found 3-to-8-fold increases in cPHB levels in diabetic rats compared to the control animals in the plasma and tissues affected by complications of diabetes—kidney, eye, sciatic nerve, and aorta. These data strongly indicate that cPHB may be an important factor in the development of diabetes, and that plasma cPHB levels may serve as a marker for the disease. Moreover, Norris et al. (2009) proposed that the high intrinsic viscosity of cPHB may play a role in raising intraocular pressure leading to glaucoma. They suggested that cPHB, adhering to filaments in the extracellular matrix, traps debris and triggers aggregation of fibers. This reduces the size of the pores and the flow of the aqueous humor through the meshwork, and thereby raises intraocular pressure.

Using a crotonic acid detection test and cPHB antibodies, Rosetta Reusch was first to demonstrate that cPHB is present in human plasma mainly together with the low-density lipoproteins and with the carrier protein albumin (Reusch et al., 1992). Reusch et al. (1992) determined cPHB levels in plasma of a random group of 24 normal adults and found that concentrations varied from 0.6 to 18.2 mg/l, with a mean of 3.5 mg/l. They further determined that cPHB is carried in lipoprotein particles and albumin—20 to 30% of cPHB is in VLDLs, intermediate density lipoproteins (IDL), and LDLs with most of the remaining 70–80% in albumin. Importantly, they found that cPHB concentrations in plasma correlated strongly with atherogenic lipid profiles. Moreover, no cPHB was associated with HDL. cPHB from ingested food may enter the circulation in the chylomicrons and VLDL similarly to cholesterol (Ramasamy, 2013; Welty, 2013). The amount of cPHB in the VLDL may be a function of diet, postprandial phase, and genetic factors. Since cPHB cannot be extracted from VLDL with CHCl_3_, it is considered to be tightly complexed to proteins. The presence of esterases or depolymerases in VLDL is indicated by the lability of cPHB in these particles (Reusch et al., 1992). As VLDL are converted to IDL and then to LDL in the VLDL-IDL-LDL cascade (Ramasamy, 2013), cPHB may be degraded to β-OHB and/or transferred to albumin which solubilizes cPHB and binds it irreversibly (Reusch et al., 1992). Presumably the cPHB-laden albumin is taken to the liver for disposal. cPHB which eludes these disposal mechanisms remains in the LDL. It appears that cPHB accumulates in the denser subgroup of LDL that is most atherogenic (Welty, 2013). It is also noteworthy that cPHB in LDL is CHCl_3_-soluble, indicating that it is no longer complexed to protein (Reusch et al., 1992). This cPHB, like cholesterol, is likely carried within the lipid core of the LDL particle, which is composed of triglycerides and phospholipids (Reusch et al., 1992; Reusch, 2012). It is this free polyester that may be harmful. As LDL travel through the circulation, some cPHB may be deposited in the arteries and act as a nucleus for the accumulation of cholesterol and other lipids, proteins, salts, etc., thus enhancing atheroma formation (Reusch, 2012). cPHB may also insert into cell membranes, making them non-specifically permeable to ions. In fact the presence of small amounts cPHB was demonstrated in mitochondria isolated from healthy bovine hearts (Seebach et al., 1994). Studies from Pavlov's group (Elustondo et al., 2013a,b; Smithen et al., 2013) addressed the potential role of cPHB in regulation of mitochondrial function. First, they revealed that endogenous cPHB plays a role in mitochondrial Ca^2+^ uptake (Smithen et al., 2013). Mitochondrial PHB depletion achieved by targeted expression of bacterial PHB hydrolyzing enzyme (PhaZ7) significantly inhibited mitochondrial Ca^2+^ uptake stimulated by ATP in intact HepG2, U87 and HeLa cells or by elevated extramitochondrial Ca^2+^in permeabilized cells. Furthermore, they (Elustondo et al., 2013a,b) demonstrated that addition of synthetic fluorophore-labeled cPHB (fluo-PHB) led to PHB accumulation specifically in mitochondria of cultured HeLa cells. Accumulation of fluo-PHB induced mitochondrial membrane potential depolarization that was delayed by cyclosporin A, de-sensitizer of the mPTP. Moreover, it was demonstrated that fluo-PHB addition caused the transient increase in cytosolic Ca^2+^ concentration in human-derived wild-type SH-SY5Y cells without any affect on mitochondrial Ca^2+^ concentration. However, addition of fluo-PHB to the PINK1 knock-out SH-SY5Y cells which have an impaired mitochondrial Ca^2+^ efflux due to the reduced activity of the mitochondrial Ca^2+^/Na^+^ exchanger (Gandhi et al., 2009) led to a significant and more sustainable accumulation of Ca^2+^ inside mitochondria. These data indicate that similar to results obtained in phospholipid bilayers of artificial vesicles (Seebach and Fritz, 1999), PHB can increase permeability of plasma and mitochondrial membrane to Ca^2+^ especially under certain pathological conditions. The exact mechanisms of the increased permeability for Ca^2+^ are unknown, however it has been proposed that PHB can act as a natural Ca^2+^ ionophore (Elustondo et al., 2013a). Consideration of PHB as a potential natural ionophore is particularly important in the light of the slow kinetics of mitochondrial ion transport discussed in Kane and Pavlov (2013). Using simple calculations, the authors concluded that the rates of ion transport in intact mitochondria are an order of magnitude lower than can be expected from the estimates based on electrophysiological data obtained in swollen mitoplasts. Specifically: (1) based on direct patch-clamp assays, the Ca^2+^ uniporter is expected to generate a current equivalent to ~50 pA per single mitochondrion (Kirichok et al., 2004), with some recent papers reporting even higher values (Jean-Quartier et al., 2012; Bondarenko et al., 2013). The estimated maximal uniporter current across the membrane of the functional, intact organelle is expected to be only about 0.1 pA. Furthermore, the uniporter Ca^2+^ flux in the mitochondria of intact cells is estimated to be in the order of 0.005 pA. The presence of slow Ca^2+^ uptake system was in fact suggested to maintain nearly normal animal developmental and physiological function in mitochondrial Ca^2+^ uniporter knock-out mice (Pan et al., 2013). Moreover, it should be noted that cPHB incorporation in membranes would also increase bilayer viscosity, which may negatively influence the performance of membrane proteins (Reusch, 2012, 2013). The fact that mitochondria with highly negative membrane potential across the inner mitochondrial membrane (ΔΨ = ~-180 mV) can accumulate fluo-PHB, which is a negatively charged polymer, suggests the existence of an active transport system for PHB. Cyclosporin A sensitivity of PHB-induced mitochondrial membrane depolarization suggests the possible involvement of PHB in activation or regulation of mPTP.

## What is inorganic polyphosphate?

**Figure 3:**
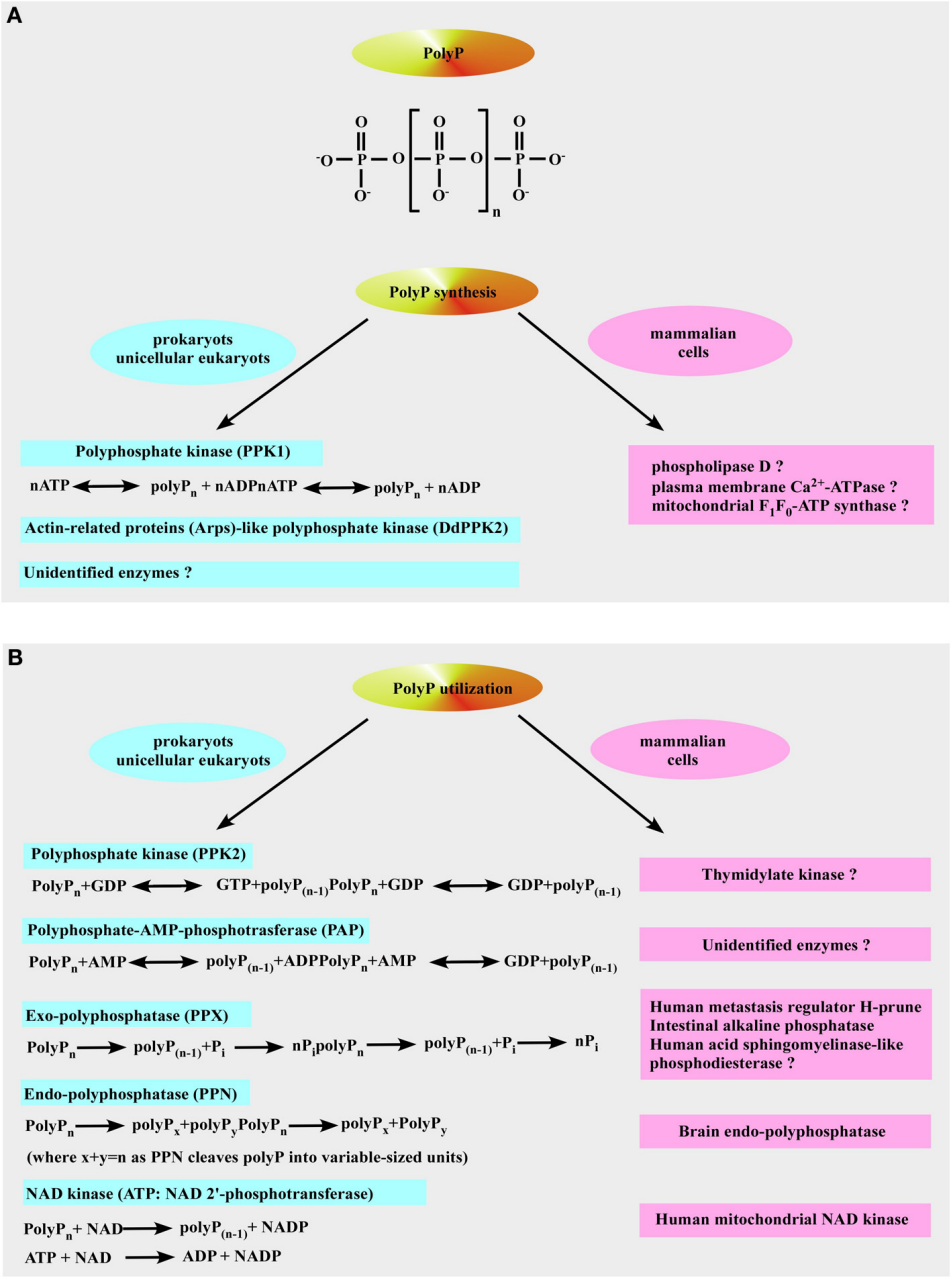
**Inorganic polyphosphate structure, formation, and utilization. (A)** The upper panel shows the structure of inorganic polyphosphate. The n represents the number of phosphate residues in the polyphosphate chain. It could vary from ten to hundreds of units. The bottom panel is a schematic representation of polyP formation by polyP-generating enzymes in prokaryotes (left) and the putative polyP-generating enzymes in mammalian cells (right). **(B)** Identified pathways and enzymes for polyP utilization in prokaryotes are shown on the left. Mammalian enzymes which resemble the function of polyP-utilizing enzymes in prokaryotes are shown on the right (see text for more details).

polyP is a linear polymer of orthophosphate (P_i_) residues linked together by high-energy phosphoanhydride bonds as in ATP (Figure 3A) (Kornberg et al., 1999). PolyP is present in all living organisms ranging from bacteria to human, and possibly even predating life of this planet (Brown and Kornberg, 2004). The length of polyP chain can vary from just a few phosphates to several thousands phosphate units long, depending on the organism and the tissue in which it is synthesized (Kornberg et al., 1999; Brown and Kornberg, 2004, 2008). PolyP was extensively studied in prokaryotes and unicellular eukaryotes by Kulaev's group in Russian Academy of Sciences (Kulaev et al., 1999a,b) and by the Nobel Prize Laureate Arthur Kornberg at Stanford University (Kornberg et al., 1999; Brown and Kornberg, 2008). In prokaryotes, polyP is synthesized primarily by polyP kinase 1 (PPK1) via transferring the terminal phosphate from ATP to the end of the growing polyP chain (Figure 3A), and this reaction is fully reversible and may allow the bacteria to synthesize ATP from stored polyP in times of starvation and environmental stress (Kornberg et al., 1999; Brown and Kornberg, 2004, 2008). Null mutants of PPK1, with low polyP levels, are deficient in survival: namely, they show deficient responses to physical-chemical stresses and predation (Brown and Kornberg, 2004, 2008). Importantly, PPK1 is not the sole polyP-generating enzyme in bacteria since null mutants of PPK1 which lack the ability to produce long-chain polyP (Crooke et al., 1994) still contained a membrane-bound short-chain polyP with 60–70 P_i_ residues (Castuma et al., 1995). Kornberg's group was the first to identify the presence of a new type of PPK (termed DdPPK2, Figure 3A) in *Dictyostelium discoideum* slime mold (Gomez-Garcia and Kornberg, 2004). Analysis of the single 43-kDa band yielded the remarkable result that the enzyme, DdPPK2, is likely a complex of three actin-related proteins: Arp1, Arp2, and an unreported Arpx that are similar to muscle actins in size and properties. The enzymatic activity of DdPPK2 is highly unusual: (i) synthesis of polyP by DdPPK2 is blocked by actin inhibitors; (ii) this particular Arp complex is an enzyme that can polymerize into an actin-like filament concurrent with its synthesis of a polyP chain in a fully reversible reaction (Spudich, 2004). However, most bacteria synthesize polyP by unknown mechanisms since recent genome screenings through the genome search engine BLAST revealed that PPK1 and PPK2 are present together in less than half of bacterial taxa and that a third of taxa have neither enzyme (Whitehead et al., 2014).

**Figure 4:**
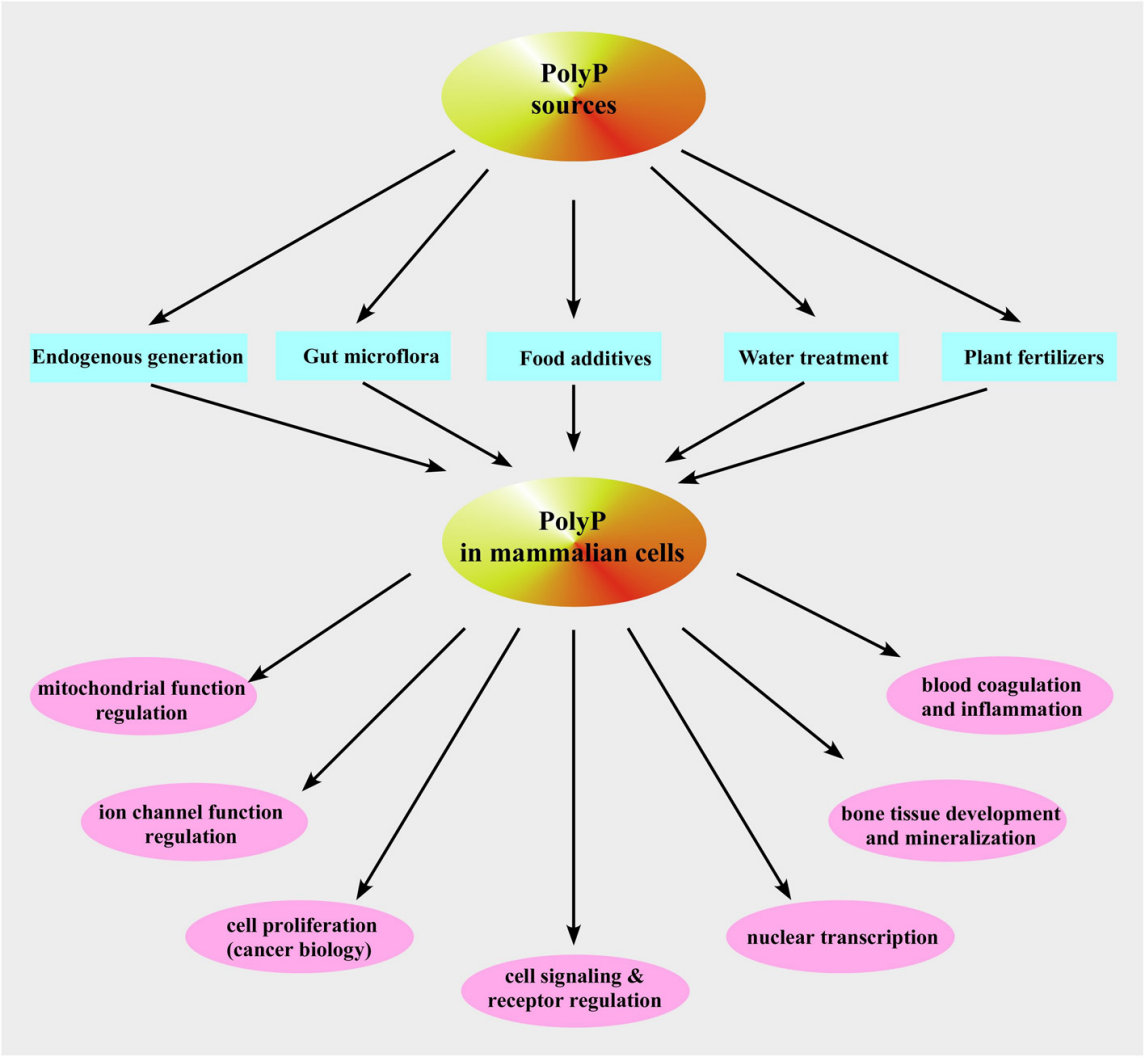
**Inorganic polyphosphate sources and functions in mammalian cells**. Shown is a schematic representation of the described pathways of polyP occurrence and functions in mammalian cells.

**Figure 5:**
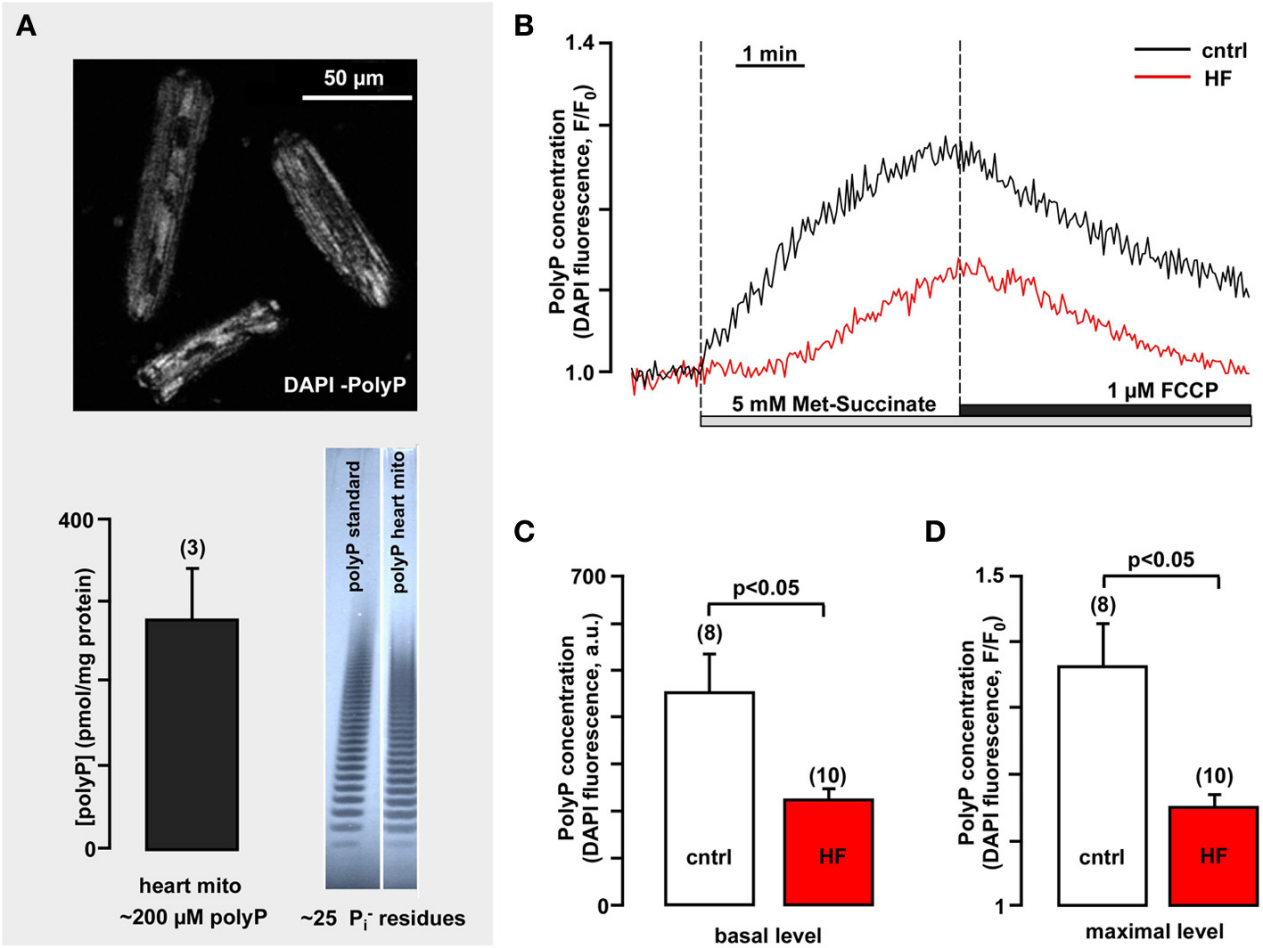
**Mitochondrial polyP concentration is highly variable in healthy and heart failure cardiomyocytes and depends on respiratory chain activity**. **(A)** The upper panel image demonstrates polyP detection in freshly isolated rabbit ventricular myocytes using DAPI as a sensor for polyP (λ_ex_ = 408 nm, λ_em_ = 552–617 nm). The bottom panel shows the average amount of polyP in rabbit heart mitochondria (left) and gel images of polyP standard and polyP sample from isolated rabbit mitochondria (right). **(B)** Original recordings of DAPI fluorescence changes in intact cardiac myocytes stimulated with 5 mM methyl-succinate followed by 5 μM FCCP from control (black) and failing myocytes (red). DAPI fluorescence represents changes in polyP concentration. **(C)** Average values of basal DAPI fluorescence in control (black) and heart failure (red) myocytes. **(D)** Average values of maximal DAPI fluorescence after methyl-succinate addition in control (black) and heart failure (red) cells. Modified with permission from Seidlmayer et al. (2012a,b).

So far, no PPK1 homolog has been identified in higher-order eukaryotes even though it is structurally similar to phospholipase D (Zhu et al., 2005) and, therefore, PPK1 exhibits potential as a novel target for chemotherapy that would affect both virulence and susceptibility to antibacterial compounds (Brown and Kornberg, 2008). Moreover, it has been demonstrated that plasma membrane Ca^2+^-ATPase from human erythrocytes may function as a polyphosphate kinase, i.e., it exhibits ATP-polyphosphate transferase and polyphosphate-ADP transferase activities (Reusch et al., 1997). The mitochondrial F_1_F_0_-ATP synthase can also contribute to polyP generation (see below and Pavlov et al., 2010; Seidlmayer et al., 2012a for details). Two bacterial enzymes [the second PPK (PPK2) and PolyP–AMP–phosphotransferases (PAP)] use polyP as a substrate. PPK2 actually resembles mammalian thymidylate kinase (Whitehead et al., 2013). PAP uses polyP as a substrate to phosphorylate AMP to ADP, an immediate precursor of ATP. PolyP is degraded by both endopolyphosphatases (PPNs) and exopolyphosphatases (PPXs) (Figure 3B). In mammalians, a long-chain endopolyphosphatase was purified from rat and bovine brain (Kumble and Kornberg, 1996), a human metastasis regulator protein H-prune was identified as a short-chain specific exopolyphosphatase (Tammenkoski et al., 2008), and mammalian intestinal alkaline phosphatase was characterized as a very active exopolyphosphatase (Lorenz and Schroder, 2001). In addition, 41% homology has been found between yeast exopolyphosphatase PP1 gene product and human acid sphingomyelinase-like phosphodiesterase (Duan, 2006; Kulakovskaya and Kulaev, 2013). In the colon, this enzyme may play anti-proliferative and anti-inflammatory roles via ceramide generation, reducing the lysophosphatidic acid formation, and inactivating the platelet-activating factor and mutations in its gene have been found in cancer cells of the intestines (Duan, 2006). Interestingly, the human protein H-prune exhibits 91% homology with the sequences of yeast exopolyphosphatase PPX1 (Kulakovskaya and Kulaev, 2013). Furthermore, in some prokaryotes [such as *Micrococcus luteus, Corynebacterium ammoniagenes* (Fillipovich et al., 2000), *Micrococcus flavus and Mycobacterium tuberculosis* (Kawai et al., 2000)] NAD kinase catalyzes phosphorylation of NAD using both ATP and polyP as phosphoryl donors (Figure 3B) while *E. Coli* NAD kinase is not able to use polyP (Kawai et al., 2001). Remarkably, human mitochondrial NAD kinase has been recently identified to have the ability to utilize both ATP and polyP as the phosphoryl donor (Ohashi et al., 2012). Neglected and long regarded a molecular fossil, polyP has a variety of significant functions in bacteria such as a (i) source of energy (Kulaev, 1979; Wood and Clark, 1988; Kulaev et al., 1999b), (ii) phosphate reservoir (Kulaev et al., 1999b), (iii) donor for sugar and adenylate kinases (Bonting et al., 1991; Hsieh et al., 1993; Phillips et al., 1993), (iv) chelator for divalent cations (Van Veen et al., 1993), (v) buffer against alkaline stress (Pick et al., 1990), (vi) regulator of development (Gezelius et al., 1973), and (vii) structural element in competence for DNA entry and transformation (Reusch and Sadoff, 1988). Even though most of polyP research has been performed in microorganisms, the presence of polyP has been demonstrated in many mammalian tissues (Figure 4) such as rodent liver, kidney, lungs, brain, and heart (Kumble and Kornberg, 1995), rabbit heart (Seidlmayer et al., 2012a,b), as well as in human granulocytes (Cowling and Birnboim, 1994), platelets (Smith et al., 2006; Smith and Morrissey, 2008a; Morrissey et al., 2012), and fibroblasts (Pisoni and Lindley, 1992). In striking contrast to microorganisms where polyP is present in millimolar (50–120 mM) concentrations, levels of 25–200 μM (it terms of P_i_ residues) were found in vast majority of mammalian tissues (Kumble and Kornberg, 1995; Seidlmayer et al., 2012b). The exceptions are platelets and mast cells which contain millimolar concentrations of polyP in electron dense granules (see below). Intracellular distribution of polyP also varies with relatively higher levels of polyP detected in nuclei and plasma membranes isolated from rat liver compared to the cytosol, mitochondria, and microsome fractions (Kumble and Kornberg, 1995). Our studies performed on mitochondria isolated from rabbit hearts detected the presence of ~200 μM (280 pmol/mg of protein) short-chain polyP with an average chain length of 25 orthophosphates (Figure 5A modified from Seidlmayer et al., 2012b). Because polyP is found in small amounts in mammalian cells, it does not serve as phosphate or energy storage (Figure 4) but is implicated in cell proliferation (Wang et al., 2003), angiogenesis (Han et al., 2007), apoptosis (Hernandez-Ruiz et al., 2006), osteoblast function (Kawazoe et al., 2004), blood clotting and inflammation (Smith et al., 2006, 2010; Smith and Morrissey, 2008a,b; Muller et al., 2009; Mutch et al., 2010a,b; Choi et al., 2011; Morrissey et al., 2012), cell bioenergetics (Pavlov et al., 2010; Seidlmayer et al., 2012a), ion channel function (Abramov et al., 2007; Kim and Cavanaugh, 2007; Zakharian et al., 2009; Seidlmayer et al., 2012b) and nuclear transcription (Jimenez-Nunez et al., 2012). These new discoveries compelled us to take a fresh look at this natural polymer that has been ignored in biochemistry textbooks for a long time.

## polyP metabolism in mammalian cells and role of mitochondria

At present very little is known about the molecular details of polyP metabolism in mammalian cells, however it has been demonstrated that newly identified human mitochondrial NAD kinase utilizes not only ATP but also polyP as the phosphoryl donor (Ohashi et al., 2012). It has been postulated for many years that only a subset of bacterial or archaeal NAD kinases exhibit high polyP-dependent NAD kinase activity, while eukaryotic NAD kinases do not (Kawai and Murata, 2008). NAD kinase is the sole NADP^+^-biosynthetic enzyme known to catalyze phosphorylation of NAD^+^ to yield NADP^+^ and plays a role in the defense against mitochondrial oxidative stress (Pollak et al., 2007). To date no mammalian polyP producing enzymes have been identified, however it has been demonstrated that polyP production in mammalian cells depends on the metabolic state of the mitochondria (Pavlov et al., 2010; Seidlmayer et al., 2012a). Experiments performed on isolated rat liver mitochondria, cultured intact cells (astrocytes, HEK 293) and rabbit cardiomyocytes demonstrated that levels of polyP were increased by substrates of the mitochondrial respiratory chain and in turn reduced by mitochondrial inhibitor (rotenone) or respiratory chain uncouplers [carbonylcyanide *p*-trifluoromethoxyphenylhydrazone (FCCP) or carbonylcyanide-m-chlorophenylhydrazone (CCCP)]. Oligomycin, an inhibitor of mitochondrial F_1_F_0_-ATP-synthase, blocked the production of poly P. These data suggest that in mammalian mitochondrial polyP production is closely related to the activity of the oligomycin-dependent F_1_F_0_-ATP synthase. However, whether or not F_1_F_0_-ATP synthase is polyP generating enzyme remains to be validated. Furthermore, enzymatic depletion of polyP from cells achieved by overexpression of the mitochondria-targeted yeast polyphosphatase (MTS-GFP-scPPX1) significantly impaired respiratory chain activity (Abramov et al., 2007) and decreased the rate of ATP production which indicates the existence of a feedback mechanism between polyP production and cell energy metabolism (Pavlov et al., 2010). These data are in agreement with earlier studies, where the turnover of polyP demonstrated dependence on the metabolic activity of cells examined (Kumble and Kornberg, 1995). Addition of radioactive ^32^**P**_i_ to cultured cells led to rapid (within minutes to 1 h) phosphate incorporation into polyP in PC12 cells while less metabolically active cells such as embryonic kidney cells and T-cells (Jurkat) showed a significantly slower or no polyP synthesis at all (Kumble and Kornberg, 1995). Furthermore, lysis of cells resulted in a loss of polyP synthetic activity. The authors postulated that polyP synthesis is an energy-dependent process which requires participation of mitochondria (Kumble and Kornberg, 1995; Kornberg et al., 1999).

We investigated the kinetics of mitochondrial polyP metabolism (Seidlmayer et al., 2012a) in intact ventricular cardiomyocytes isolated from control rabbits and animals with HF combined aortic insufficiency and stenosis model (Dedkova et al., 2013). The relative changes in levels of polyP were measured using the fluorescent probe DAPI, with a protocol optimized specifically for polyP detection (Aschar-Sobbi et al., 2008; Seidlmayer et al., 2012b). As demonstrated in Figure 5B addition of membrane permeable methyl-succinate—the substrate of the complex II of the mitochondrial respiratory chain—resulted in an increase in DAPI fluorescence by 36 ± 8% (*n* = 8), indicating significant stimulation of the production of mitochondrial polyP (Figures 5B,C). On the other hand uncoupling of respiration with FCCP decreased DAPI fluorescence by 29 ± 4% (*n* = 8) presumably due to the stimulation of polyP hydrolysis. This indicates that polyP concentration in cardiac myocytes is variable and depends on levels of energy substrates and the degree of coupling of the mitochondrial respiratory chain. Moreover, we found that polyP metabolism was significantly suppressed in mitochondria of HF myocytes. Addition of methyl-succinate caused only a moderate increase in DAPI fluorescence (16 ± 2%, *n* = 10) (Figures 5B,C). Also, the basal polyP levels were significantly lower in conditions of HF (224 ± 21 a.u. in HF vs. 453 ± 80 in control) (Figure 5D). This observation raises the intriguing possibility that similarly to bacteria, polyP production in mammalian cells is directly linked to changes in cell metabolism and environment and that diminished polyP synthesis observed in HF myocytes results from the complex remodeling processes during cardiac hypertrophy and HF. A recent study (Gray et al., 2014) determined that in *E. Coli* bacteria polyP acts as an efficient protein chaperon which stabilizes proteins *in vivo*, diminishes the need for other chaperone systems to survive proteotoxic stress (temperature, low pH, oxidants) conditions, and protects a wide variety of proteins against stress-induced unfolding and aggregation. It has been demonstrated that wild type *E. coli* stains generated significant amounts of polyP in response to oxidative stress. This polyP accumulation played a critical role in *E. coli* defense against stress since PPK mutant stains which are not able to produce polyP displayed decreased survival and elevated levels of protein aggregates compared to wild-type strains in response to oxidant or heat stress. PPK mutants showed increased activation of heat shock response, consistent with them suffering from more protein damage upon a similar stress compared to the wild-type. Importantly, we reported a significant increase in reactive oxygen species (ROS) generation and cell death in polyP-depleted ventricular myocytes exposed to simulated ischemia-reperfusion (Seidlmayer et al., 2013) suggesting a protective role of polyP in conditions associated with increased oxidative stress. Furthermore, cell-free experiments revealed that polyP inhibits the aggregation of chemically and heat-denaturated luciferase and citrate synthase, previously established protein chaperon substrates (Gray et al., 2014). PolyP maintained these substrates in a refolding competent state, which required the assistance of the canonical ATP-dependent 70 kilodalton heat shock protein (Hsp70)/DnaK machine. Utilizing standard *in vitro* chaperone assays, it was demonstrated that the presence of 1 μM polyP was sufficient to significantly reduce thermal luciferase aggregation while 100 μM polyP completely prevented aggregate formation. Millimolar concentrations, however, were required to inhibit oxidant or heat-induced aggregation of citrate synthase. Moreover, long-chain polyP polymers (with 130–300 phosphate residues) were more effective as chaperones compared to short chain polymers (~14 phosphate residues). These experiments provide evidence that polyP exerts chaperone activity in concentration- and length-dependent manner and that this activity varies with different substrates. The finding that polyP has stress-protective chaperone activities that resemble the activity of small heat shock proteins is very exciting, however additional research is required to determine the mechanisms of protein aggregation prevention by polyP and the protein targets of polyP in mammalian cells.

## Inorganic polyP and activation of the mitochondrial permeability transition pore (mPTP)

**Figure 6:**
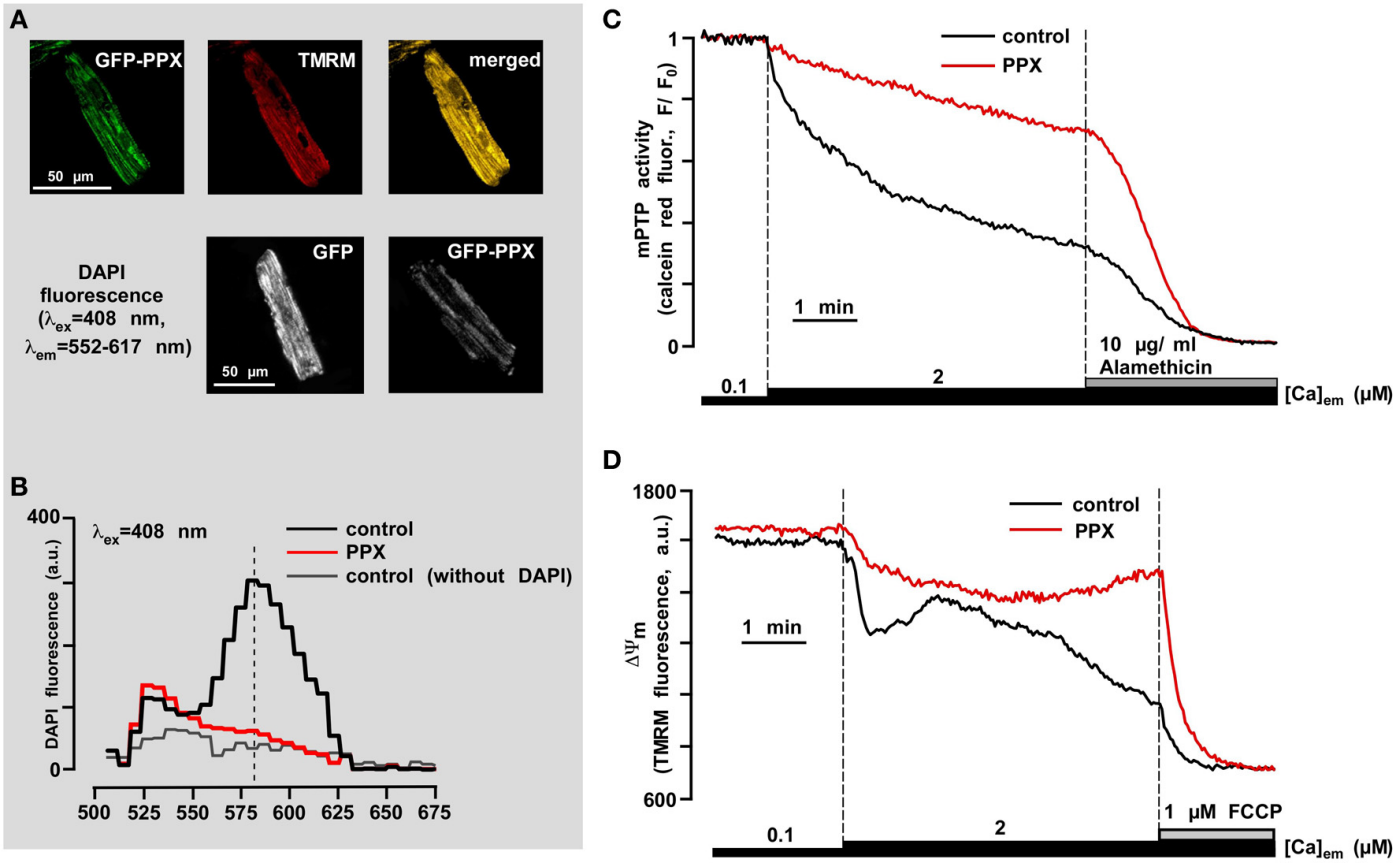
**PolyP depletion prevents opening of the permeability transition pore induced by mitochondrial Ca^2+^ overload**. **(A)** The upper panel shows co-localization of GFP-PPX signal with mitochondria. TMRM was used as a mitochondrial signal and the degree of overlay is presented in shades of yellow in the merged image. The bottom panel shows the decrease in DAPI fluorescence in polyP-depleted cells. **(B)** Fluorescence spectrum of DAPI (5 μM) loaded myocytes expressing control GFP (black), PPX (red), and control GFP cells not loaded with DAPI (gray). **(C)** Original recordings of mPTP opening using calcein red release from mitochondria of permeabilized control (black) and polyP-depleted (PPX expressing, red) myocytes. After permeabilization cells were exposed to 2 μM Ca^2+^ and 10 μg/ml alamethicin was added at the end of the experiment to achieve the maximal calcein red release from mitochondria. **(D)** Original recordings of mitochondrial membrane potential (ΔΨ_m_) with the voltage-sensitive dye TMRM in permeabilized cells upon elevation of the [Ca^2+^]_em_ from 0.1 to 2 μM, and subsequent addition of 1 μM FCCP in control (black) and polyP-depleted (PPX expressing, red) myocytes. Modified with permission from Seidlmayer et al. (2012b).

One of the most intriguing and least intuitive roles of polyP is its involvement in membrane ion transport. In 1988 Reusch and Sadoff, using bilayer techniques, demonstrated that genetically competent *E. coli* bacteria contain an ion channel formed by a complex of polyP and PHB (Reusch and Sadoff, 1988). The channel formed by polyP/Ca^2+^/PHB interaction was selective for cations with a preference for Ca^2+^(Reusch and Sadoff, 1988; Reusch et al., 1995). Later a similar polyP/Ca^2+^/PHB channel was isolated from rat liver mitochondria (Pavlov et al., 2005b). Interestingly, in addition to the cation selective conductance state this mitochondrial complex also demonstrated a high-conductance, weakly-selective, voltage-dependent state. These properties in many ways reflected the behavior of the mPTP as seen in patch-clamp studies of native mitochondrial membranes (Kinnally et al., 1991; Szabo and Zoratti, 1991). Interestingly, the polyP/Ca^2+^/PHB channel of bacterial origin also has this high conductance state (Pavlov et al., 2005a) and the transition of the channel into a high conductance state would most likely be deleterious for bacterial organisms, raising the question whether most of the time the bacterial channel is either closed or is in the low conductance cationic state. The different bacterial conductance states are reminiscent of conductance states proposed for the mPTP (Ichas et al., 1997; Huser et al., 1998; Ichas and Mazat, 1998). The parallels between bacteria and mitochondria also suggest that similar cationic channels may play a role in normal mitochondrial function. In support of such notion the polyP/Ca^2+^/PHB complex has been detected in various eukaryotic organisms and cellular compartments suggesting a potential physiological role (Reusch, 1989). Currently, the direct test whether a polyP/Ca^2+^/PHB complex indeed forms the pore part of the mPTP in intact mitochondria remains an experimental challenge. Nonetheless, the idea that the presence of polyP in intact mitochondria is an essential condition for mPTP opening remains an intriguing hypothesis. Indeed, it was shown that mitochondria of cultured cells with reduced levels of polyP are more resistant toward Ca^2+^-induced mPTP opening (Abramov et al., 2007). In our work performed in rabbit ventricular cardiomyocytes we demonstrated that polyP depletion with MTS-GFP-scPPX1 overexpression effectively prevented Ca^2+^-induced mPTP opening (Figures 6A,B modified from Seidlmayer et al., 2012b). In contrast to non-excitable cells (Abramov et al., 2007), polyP depletion did not affect the ability of mitochondria to accumulate Ca^2+^, however significantly increased the resistance of cardiac mitochondria to open mPTP (Figure 6C) and prevented Ca^2+^-induced loss of mitochondrial membrane potential (Figure 6D) indicating that polyP is a potent activator of Ca^2+^-induced mPTP. On the other hand, when mPTP activity was monitored in conditions of simulated ischemia-reperfusion accompanied by massive ROS generation, polyP depletion was not able to prevent mPTP opening and cell death during reperfusion. In fact, as we demonstrated earlier (Seidlmayer et al., 2013), ROS generation and cell death was significantly increased under conditions of ischemia-reperfusion in polyP-depleted cells. We found different modes in mPTP activity during ischemia and reperfusion, and that these modes were affected differently by polyP. In agreement with our data obtained on permeabilized cells (Seidlmayer et al., 2012b), polyP depletion prevented Ca^2+^-induced low conductance mPTP mode observed during ischemia, however it did not affect ROS-induced mPTP opening in the high-conductance mode during reperfusion. These exciting findings indicate that polyP has a dual effect on mPTP activity—promoting the transient opening of Ca^2+-induced^ mPTP opening which can prevent mitochondria from Ca^2+^ overload. On the other hand, polyP was required for protection against oxidative stress-induced mPTP opening and cell death. It is unclear at this point, whether this effect of polyP was related to the recently discovered chaperone activity of polyP or the direct effect of polyP on mPTP. Recent data suggest that dimers of the of F_1_F_0_-ATP synthase can form channels with characteristics similar to the mPTP (Giorgio et al., 2013), however the molecular details of channel formation by F_1_F_0_-ATP synthase remain unclear. Particular attention has been brought to the subunit c of the F_1_F_0_-ATP synthase as a potential component of the mPTP (Azarashvili et al., 2014; Bonora et al., 2013). Interestingly, an interaction of polyP/Ca^2+^/PHB complex with subunit c of F_1_F_0_-ATP synthase was reported back in 2005 (Pavlov et al., 2005b), and therefore it is possible that polyP could provide a fine tuning of mPTP regulation or actually mediate Ca^2+^ transfer through mPTP.

## polyP is a link between human gastrointestinal tract bacteria, β-OHB and cardiovascular Health

It has recently been discovered that human gastrointestinal tract bacteria (probiotics) produce polyP, and that polyP is responsible for probiotic actions that protect the intestinal epithelia from oxidant stress and improve epithelial injury due to excess inflammation (Segawa et al., 2011). It has been shown that polyP specifically binds to integrin β1, and inhibition of integrins or p38 MAPK pathway eliminates the protective effect of polyP on intestinal epithelia (Segawa et al., 2011). P38 MAPK is a class of mitogen-activated protein kinases responsive to stress stimuli, such as cytokines, ultraviolet irradiation, heat shock and osmotic shock, and is involved in cell differentiation and apoptosis. Indeed, protective effect of polyP was associated with its ability to induce expression of the cytoprotective heat shock protein 27 (Hsp27) in these cells (Segawa et al., 2011) and to decrease staurosporine-induced apoptosis as evidenced by the inhibition of caspase-9 and caspase-3 activation. Althogether, these results suggest that polyP develops a robust intestinal barrier function through interaction with integrin β1, followed by the p38 pathway activation. Intriguingly, recent studies (Lam et al., 2012; Gan et al., 2014) demonstrated that probiotic administration attenuates myocardial infarction following ischemia-reperfusion injury and myocardial hypertrophy and HF following myocardial infarction in the rat. Lam and coworkers (Lam et al., 2012) provided the first evidence that probiotics may be cardioprotective by showing that administration of a commercially-available beverage containing the probiotic *Lactobacillus plantarum* 299 v 24 h before subjecting rats to 30 min of cardiac ischemia and 2 h reperfusion, produced a 27% reduction in infarct size and improved reperfusion by 35%. In the study of Gan et al. (2014), rats were subjected to 6 weeks of sustained coronary artery occlusion and administered the probiotic *Lactobacillus rhamnosus* GR-1 or placebo. They found that animals administered GR-1 exhibited a significant attenuation of left ventricular hypertrophy and improved hemodynamic parameters. Serial echocardiography revealed significantly improved left ventricular parameters throughout the 6 week follow-up period including a marked preservation of left ventricular ejection fraction as well as fractional shortening. Beneficial effects of GR-1 were still evident in those animals in which GR-1 was withdrawn at 4 weeks suggesting persistence of the GR-1 effects following cessation of therapy. Investigation of mechanisms showed a significant increase in the leptin to adiponectin plasma concentration ratio in rats subjected to coronary ligation which was abrogated by GR-1. Metabonomic analysis showed differences between sham control and coronary artery ligated hearts particularly with respect to preservation of myocardial taurine levels. These studies suggest that gut microbiota can modulate cardiovascular disease possibly due to alterations in the production of gut-derived hormones which exert cardiovascular effects. Specifically, both studies detected a significant decrease in leptin production in probiotic-treated animals. PolyP levels were not measured in these studies, however, it is plausible to speculate that polyP produced by gut microbiota could also exerts a cardioprotective role for the host organism. Interestingly, germ-free mice which were born and raised in sterile gnotobiotic isolators, had a significantly reduced heart weight (Crawford et al., 2009) compared to those of normally colonized mice. Metabolic and physiological studies revealed that functional parameters (heart rate, hydraulic work, mitochondrial morphology, number, and respiration, plus ketone body, fatty acid, and glucose oxidation) of the hearts of germ-free mice were relatively normal in fed conditions. However, a significant decrease in hepatic ketogenesis and levels of circulating β-OHB was detected under fasting conditions in germ-free mice with a compensatory increase in cardiac glucose oxidation (Crawford et al., 2009). The reduction in heart size and alterations of myocardial metabolism were reversed in germ-free mice maintained on a ketogenic diet or following microbial colonization (Crawford et al., 2009). Altogether, the data demonstrate the existence of a link between gut microbiota and cardiovascular health, however the exact roles of polyP and β-OHB in cardiovascular health and disease remain to be determined. It is intriguing to speculate that polyP produced by gut microbiota can modulate signaling cascades and ketone body metabolism. Importantly, mammalian intestinal alkaline phosphatase has been described as a very active exopolyphosphatase (Lorenz and Schroder, 2001), which can split long-chain polyP produced by gut microbiota into short-chain polyP and phosphate. The short-chain polyP could be efficiently absorbed in human intestinal tract and excreted in the urine (Karp et al., 2012). A recent study (Karp et al., 2012) compared the effect of polyP and phosphate supplementation in healthy women 19–31 years of age and revealed that both monophosphate and polyP induced an increase in serum parathyroid hormone and serum/urine phosphate levels, while only polyP led to a significant decrease in urinary Ca^2+^ levels. This decrease in Ca^2+^ levels most likely was due to enhanced Ca^2+^ binding to polyP in intestine and decreased Ca^2+^ absorption (Zemel and Linkswiler, 1981). In mammals, circulating inorganic phosphate and polyP (complexes with Ca^2+^) serve to support extracellular mineralization, which appears to furthermore depend on the concerted expression of collagen type 1 and tissue-non-specific alkaline phosphatase (Murshed et al., 2005). To control mineralization and cellular delivery, extracellular phosphate levels and total body phosphate content are tightly regulated by a number of hormones, including parathyroid hormone, 1,25-dihydroxy-vitamin D, and fibroblast growth factor-23 (*FGF23*), and serum phosphate feeds back to regulate these factors in an endocrine fashion (Civitelli and Ziambaras, 2011): high phosphate increases the secretion of parathyroid hormone and *FGF23*, while low phosphate stimulates the synthesis of 1,25-dihydroxy-vitamin D. Hypophosphatemia leads to demineralization of the skeleton (osteomalacia), whereas hyperphosphatemia is an important risk factor for the development of vascular calcifications (Rutsch et al., 2011). Interestingly, it has been shown recently that polyP actually stimulates *FGF23* expression through activation of fibroblast growth factor receptor (Sun et al., 2012), and promotes bone mineralization (Hacchou et al., 2007). Moreover, while polyP of different chain length (short, medium, and long) stimulated *FGF23* expression, polyP with long chains actually led to the increased lactic acid accumulation and cell death (Sun et al., 2012). Furthermore, high levels of circulating *FGF23* are linked to the increased incidents of pathological cardiovascular events (Arnlov et al., 2013).

## Roles of polyP and β-OHB in cell proliferation—implications for cancer biology

It has been demonstrated that addition of polyP to human plasma cells produced an unexpected inhibition of immunoglobulin secretion and stimulation of apoptosis. PolyP induced apoptosis specifically in human plasma cells, myeloma (malignant plasma cells) cell lines, primary myeloma cells, and B lymphoid cell lines (Hernandez-Ruiz et al., 2006). Normal B cells, T cells, total blood mononuclear cells, and non-lymphoid cell lines were not affected by polyP. In the U266 myeloma cell line, polyP induced externalization of phosphatidylserine, activation of caspase-3, and arrest of the cell cycle. The protective effects of interleukin-6 did not overcome the polyP-induced apoptosis. This study, however, used very high (3–6 mM) concentrations of polyP with various chain lengths (25, 45, and 75 P_i_ residues), and detected that in these concentrations polyP effectively inhibited immunoglobulin secretion, initiated apoptosis and reduced cell survival of the U266 myeloma cell line. Since total peripheral blood mononuclear cells, T cells, B cells, and non-lymphoid cell lines were not affected, it was suggested that polyP could potentially be useful in the design of new antimyeloma drugs. Also, polyP could contribute in part to maintaining low levels of plasma cells in blood circulation (Jimenez-Nunez et al., 2012).

Another study (Han et al., 2007) demonstrated that polyP effectively blocked *in vivo* pulmonary metastasis of B16BL6 cells by suppression of neovascularization, whereas it did not affect proliferation or adhesion to extracellular matrix proteins. PolyP not only inhibited bFGF (basic fibroblast growth factor)-induced proliferation and ERK (extracellular-signal-regulated kinase)/p38 MAPK (mitogen-activated protein kinase) activation of human endothelial cells, but also blocked the binding of bFGF to its cognate cell surface receptor. Furthermore, polyP inhibited bFGF-induced *in vitro* and *in vivo* angiogenesis, suggesting that polyP possesses an anti-angiogenic activity. Since neovascularization is essential for tumor metastasis, these findings indicate that polyP has an *in vivo* anti-metastatic activity via its anti-angiogenic activity.

Interestingly, ketogenic diet lowered the serum ratio of IGF/IGF-binding protein 3 in mice with positive effects on metabolic syndrome and cancer risk (Freedland et al., 2008). Also, ketogenic diet (and elevated β–OHB) increased AMPK in mice, inhibiting the mTOR/Akt signaling pathway (McDaniel et al., 2011). However, polyP was also found to stimulate mTOR signaling and possibly cancer growth (Wang et al., 2003). In this study, polyP depletion achieved by overexpression of yeast exopolyphosphatase *PPX1* gene inhibited proliferation of the human breast carcinoma cell line MCF-7 via mTOR signaling cascade. These conflicting results definitely should stimulate further research to determine the exact roles of polyP and β–OHB in cell growth and cancer biology. Recently, human metastasis regulator protein H-prune was identified as a short-chain specific polyP hydrolase (Tammenkoski et al., 2008). Long-chain polyP (>25 phosphate residues) were converted more slowly, whereas pyrophosphate and nucleoside triphosphates were not hydrolyzed. Notably, the exopolyphosphatase activity of H-prune was suppressed by long-chain polyphosphates and pyrophosphate, which could be potential physiological regulators. Clearly, the chain length of polymer determines the physiological role of polyP making it either a friend or foe.

## Role of polyP in bone tissue development and mineralization

The vertebrate skeleton is predominately composed of bone, a mineralized tissue that consists of type-1 collagen, non-collagenous proteins and a calcium phosphate mineral known as apatite (Omelon et al., 2009). Unlike invertebrate skeletons or protective shells, the vertebrate skeleton is continually rebuilt, repaired and resorbed during growth, normal remodeling, and recovery from traumas and diseases. Even though the role of polyP in the modulation of bone mineralization was suggested nearly 15 years ago (Leyhausen et al., 1998), only recently considerable advances were made in the study of polyP contribution to bone tissue development (Kawazoe et al., 2004; Hacchou et al., 2007; Omelon et al., 2009; Morimoto et al., 2010; Usui et al., 2010). It has been shown that polyP is present abundantly in normal human osteobalsts, at bone-resorbing osteoclastic sites, in the proliferating and hypertrophic zone chondrocytes, in the hypertrophic zone matrix, and in unmineralized osteoid (Shiba et al., 2003; Kawazoe et al., 2008). Moreover, polyP enhanced alkaline phosphatase activity and expression of osteopontin and osteocalcin genes in MC3T3-E1 osteoblastic cells suggesting that polyP promotes calcification in these cells (Kawazoe et al., 2004). Interestingly, polyP was detected in electron-dense Ca^2+^-rich granules localized at sites of bone resorption (Omelon et al., 2009). The size of these dense granules was larger (in the range of a few thousand angstroms) than previously reported for mitochondrial electron-dense clusters in mineralizing osteoid (ranging from 400 to 1000 angstroms) (Landis et al., 1977) The authors (Omelon et al., 2009) put together a hypothesis that apatite mineral dissolution in the osteoclast resorption zone increases the concentrations of free P_i_ and Ca^2+^. The mitochondria may scavenge the P_i_ and condense it into polyP that may also sequester Ca^2+^. Amorphous granules containing total concentrations of Ca^2+^ and P_i_ higher than the saturation of apatite are formed and may be transported out of the osteoclast. The granules may be transported to or produced within the osteoblasts that build new bone. The osteoblasts may embed the granules in osteoid (new, unmineralized bone). The reaction of these granules with alkaline phosphatase (present at the membrane of osteoblasts) cleaves P_i_ from polyP and would increase the free P_i_ concentration and release any sequestered Ca^2+^. The increase in free Ca^2+^ and P_i_ could exceed the saturation for apatite and result in apatite mineral formation. Most recently (Tsutsumi et al., 2014), it was demonstrated that polyP promotes MC3T3-E1 cell maturation from a resting state to an active blastic cell phase which implies that polyP can be an effective material for bone regeneration. The enzyme responsible for polyP synthesis in bone tissue cells has not been identified and the signal molecules determining separate stages of this process are unknown. Fascinating, *in vivo* experiments demonstrated that artificial introduction of polyP into the bone growth or regeneration zone accelerates these processes (Grynpas et al., 2002; Pilliar et al., 2007; Shanjani et al., 2007; Doi et al., 2014). The finding that polyP induces osteoblastic differentiation and bone mineralization creates a basis for development of novel polyP-containing drugs for bone disease treatment and new polyP-containing materials for bone substitution.

## Role of polyP in blood coagulation and inflammation

It has been known for many years that polyP accumulates in many infectious microorganisms (Kornberg et al., 1999) and therefore could play a significant role in inflammation. Microbial polyP (with chain length of more than 500 P_i_ residues), which is highly pro-coagulant, may function in host responses to pathogens (Chuang et al., 2013). Of particular interest to hematology, it has been demonstrated that polyP is a major component of dense granules of human platelets (Ruiz et al., 2004) and acidocalcisomes of mast cells (Moreno-Sanchez et al., 2012), where millimolar levels (in terms of P_i_ residues) of short chain polyP (~70–75 phosphate units in platelets and 60 phosphate units in mast cells) were detected. Like acidocalcisomes, human platelets dense granules are spherical, acidic, electron-dense, and contain large amounts of calcium and potassium in addition to polyP (Ruiz et al., 2004). Both human platelets and mast cells secrete polyP upon activation which indicates that polyP could be an important mediator of their pro-inflammatory and pro-coaguant activities. Indeed, studies from Morrissey's lab and others have shown that polyP is a potent modulator of the blood clotting cascade, acting as a pro-hemostatic, pro-thrombotic and pro-inflammatory agent depending on its polymer size and location (Smith et al., 2010). PolyP may represent at least one of the long-sought patho-physiologic activators of the contact pathway of blood clotting, and its actions may also help to explain previously unexplained abilities of activated platelets to enhance plasma clotting reactions. Targeting polyP with phosphatases interfered with pro-coagulant activity of activated platelets and blocked platelet-induced thrombosis in mice (Muller et al., 2009). Addition of polyP restored defective plasma clotting of Hermansky–Pudlak Syndrome patients, who lack platelet polyP (Muller et al., 2009). Remarkably, both Hermansky–Pudlak (HPS) and Chediak–Higashi (CHS) syndromes have in common dense granule deficiency and bleeding tendency (Salles et al., 2008), which is congruent with the presence of poly P in dense granules (Ruiz et al., 2004) and its role in blood clotting (Smith et al., 2006). The data identify polyP as a new class of mediator having fundamental roles in platelet-driven pro-inflammatory and pro-coagulant disorders. PolyP acts via several points in the clotting cascade: (i) it triggers clotting via the contact pathway (Smith et al., 2006, 2010; Muller et al., 2009), (ii) it accelerates factor V activation (Smith et al., 2006), (iii) it enhances fibrin clot structure (Smith and Morrissey, 2008b; Mutch et al., 2010a), and (iv) it accelerates factor XI back-activation by thrombin (Mutch et al., 2010b; Choi et al., 2011). It was demonstrated that very long polymers (with more than 500 P_i_ residues, such as those present in microorganisms) were required for optimal activation of the contact pathway, while shorter polymers (with ~100 P_i_ residues, similar to the polymer lengths released by platelets) were sufficient to accelerate factor V activation and abrogate the anticoagulant function of the tissue factor pathway inhibitor. Optimal enhancement of fibrin clot turbidity by polyP required polyP with more than 250 P_i_ residues. Therefore, polyphosphate of the size secreted by platelets is very efficient at accelerating blood clotting reactions but is less efficient at initiating them or at modulating clot structure (Smith et al., 2010). PolyP may prove useful as a hemostatic agent to control bleeding, and conversely, polyP antagonists might be beneficial as anti-thrombotic/anti-inflammatory agents with reduced bleeding side effects. Several polyphosphate inhibitors have been identified recently and their effectiveness was tested *in vitro* and *in vivo* (Smith et al., 2012). Polyphosphate inhibitors were anti-thrombotic in mouse models of venous and arterial thrombosis and blocked the inflammatory effect of polyphosphate injected intra-dermally in mice. This provides proof of principle for polyphosphate inhibitors as anti-thrombotic/anti-inflammatory agents *in vitro* and *in vivo*, with a novel mode of action compared with conventional anticoagulants (Smith et al., 2012). However, the detailed molecular mechanisms by which polyP modulates blood clotting reactions still remain to be elucidated (Morrissey, 2012).

## polyP regulates innate immunity by modulating inos expression in macrophages– signaling roles of polyP

It has been proposed recently that endogenous polyP may serve as an intercellular signaling molecule in innate immunity (Harada et al., 2013). As we discussed above, both platelets and mast cells store polyP in their granules and secrete it into the extracellular space when the cells are activated (Ruiz et al., 2004; Moreno-Sanchez et al., 2012). Moreover, it has been shown that parasites such as *Trypanosoma cruzi (T. cruzi)* contain polyP within molar levels in acidocalcisomes which is essential for the parasite to resist the stressful conditions in the host and to maintain a persistent infection (Galizzi et al., 2013). Interestingly, infection with *T. cruzi* leads to the development of chronic Chagas heart disease characterized by autonomic nervous system derangements, microvascular disturbances, parasite-dependent myocardial infection, and immune-mediated myocardial injury (Marin-Neto et al., 2007). Despite nearly one century of research, the pathogenesis of chronic Chagas cardiomyopathy is incompletely understood (Marin-Neto et al., 2007). Tightly packed polyP may be released into the extracellular space and affect the function of macrophages, thereby serving as an immune modulator. Indeed, the recent study (Harada et al., 2013) demonstrated that polyP suppressed inducible nitric oxide synthase (iNOS) expression and nitric oxide (NO) release induced by lipopolysaccharide (LPS), a cell wall component of Gram-negative bacteria, in mouse peritoneal macrophages. In contrast, polyP did not affect the LPS-induced release of TNF, another inflammatory mediator. Using polyP of various chain lengths (14, 60, and 130 P_i_ residues) it was demonstrated that polyP with longer chains (130 P_i_ residues) was more potent than those with shorter chains in suppressing LPS-induced iNOS expression. It has been shown that polyP suppressed LPS-induced iNOS expression by down-regulating the level of mRNA expression, however the detailed mechanisms are currently unknown. LPS-induced extracellular Ca^2+^ influx enhances iNOS expression in macrophages (Kim et al., 2004; Zhou et al., 2006). PolyP, a negatively charged polyanion, may bind with extracellular Ca^2+^, inhibit LPS-induced Ca^2+^ influx, and result in a decreased expression of iNOS mRNA (Harada et al., 2013). On the other hand, extracellular polyP has been recently reported to modulate or activate receptors on the plasma membrane, such as the FGF, integrin, and P2Y_1_ receptors (Shiba et al., 2003; Segawa et al., 2011; Holmstrom et al., 2013). Potentially, polyP may regulate iNOS expression via modulation of these receptors.

## Conclusions

The main objective of this review was to emphasize the importance of ketone body β-OHB, its natural polymer PHB, and inorganic polyP in cardiovascular physiology and other diseases. While physicians were always taught that elevation in β-OHB levels is undesired, recent studies indicate that a mild elevation in β-OHB levels could be actually beneficial in certain physiological situations, and can modulate the important signaling cascades involved in cell growth, proliferation and defense against oxidative stress. Two ancient polymers, PHB and polyP, have proved to be involved in regulation of protein channels in plasma membrane and mitochondria, and should not be discarded as “relics” from Prebiotic times.

### Conflict of interest statement

The authors declare that the research was conducted in the absence of any commercial or financial relationships that could be construed as a potential conflict of interest.

## Abbreviations

AcAc: acetoacetate
AF: atrial fibrillation
BDH: D-β-hydroxybutyrate dehydrogenase
β-OHB: β-hydroxybutyrate
cPHB: complexed poly-β-hydroxybutyrate
FFAR3: free fatty acid receptor 3
FOXO3a: Forkhead box O3a
GPCRs: G-protein-coupled receptors
IDL: intermediate density lipoproteins
HDACs: class I histone deacetylases
HF: heart failure
HMG-CoA: β-hydroxy-β-methylglutaryl-CoA
LCKD: low carbohydrate ketogenic diet
LDL: low density lipoproteins
LFI: low flow ischemia
PHB: poly-β-hydroxybutyrate
polyP: inorganic polyphosphate
SCOT: succinyl-CoA:3-oxoacid-CoA transferase
TCA: tricarboxylic acid
VLDL: very low density lipoproteins.
